# Robust high-throughput assays to assess discrete steps in ubiquitination and related cascades

**DOI:** 10.1186/s12860-020-00262-5

**Published:** 2020-03-30

**Authors:** Gabriel Fenteany, Paras Gaur, Gaurav Sharma, Lajos Pintér, Ernő Kiss, Lajos Haracska

**Affiliations:** 1 HCEMM-BRC Mutagenesis and Carcinogenesis Research Group, Institute of Genetics, Biological Research Centre, Szeged, Temesvári krt. 62, Szeged, 6726 Hungary; 2 Doctoral School of Biology, Faculty of Science and Informatics, University of Szeged, Közép fasor 52, Szeged, 6726 Hungary; 3 Visal Plus Ltd., Temesvári krt. 62, Szeged, 6726 Hungary

**Keywords:** Ubiquitination, Ubiquitin-like proteins, Post-translational modifications, E1 ubiquitin-conjugating enzymes, E2 ubiquitin-conjugating enzymes, E3 ubiquitin ligases, Step-specific assays and evaluation, High-throughput screening

## Abstract

**Background:**

Ubiquitination and ubiquitin-like protein post-translational modifications play an enormous number of roles in cellular processes. These modifications are constituted of multistep reaction cascades. Readily implementable and robust methods to evaluate each step of the overall process, while presently limited, are critical to the understanding and modulation of the reaction sequence at any desired level, both in terms of basic research and potential therapeutic drug discovery and development.

**Results:**

We developed multiple robust and reliable high-throughput assays to interrogate each of the sequential discrete steps in the reaction cascade leading to protein ubiquitination. As models for the E1 ubiquitin-activating enzyme, the E2 ubiquitin-conjugating enzyme, the E3 ubiquitin ligase, and their ultimate substrate of ubiquitination in a cascade, we examined Uba1, Rad6, Rad18, and proliferating cell nuclear antigen (PCNA), respectively, in reconstituted systems. Identification of inhibitors of this pathway holds promise in cancer therapy since PCNA ubiquitination plays a central role in DNA damage tolerance and resulting mutagenesis. The luminescence-based assays we developed allow for the quantitative determination of the degree of formation of ubiquitin thioester conjugate intermediates with both E1 and E2 proteins, autoubiquitination of the E3 protein involved, and ubiquitination of the final substrate. Thus, all covalent adducts along the cascade can be individually probed. We tested previously identified inhibitors of this ubiquitination cascade, finding generally good correspondence between compound potency trends determined by more traditional low-throughput methods and the present high-throughput ones.

**Conclusions:**

These approaches are readily adaptable to other E1, E2, and E3 systems, and their substrates in both ubiquitination and ubiquitin-like post-translational modification cascades.

## Background

Post-translational modification of proteins through ubiquitination controls not only protein degradation by the proteasome but also myriad other processes. Ubiquitin and related ubiquitin-like proteins (UBLs) serve as tags and docking sites for protein–protein interactions that regulate a staggering array of biological pathways. Modulating various steps of ubiquitination and UBL post-translational modification pathways with small-molecule inhibitors or activators/enhancers has considerable research and therapeutic potential. One such function for ubiquitin and UBLs is in the dynamic control of the DNA damage repair and tolerance machinery. DNA damage tolerance pathways evolved to circumvent the fact that DNA sites altered by chemical insult, oxidation, photodamage, or irradiation that have not been repaired by other DNA repair mechanisms (such as base and nucleotide excision or mismatch repair) cause the stalling of the DNA replication fork. Replicative DNA polymerases are unable to copy damaged DNA. There are two general pathways of DNA damage tolerance: translesion DNA synthesis (TLS) and template switching. However, TLS is inherently prone to introducing point mutations, whereas template switching can result in chromosomal rearrangements. DNA damage tolerance can thus lead to genetic alterations, oncogenesis, formation of secondary tumors after treatment with DNA-damaging therapeutics, drug resistance, and other pathologies, with strong evidence that inhibition of the DNA damage response can lead to reversal of drug resistance in and apoptotic death of a range of different cancer cell types (reviewed in refs. [1–6]).

The ubiquitination of proliferating cell nuclear antigen (PCNA), a sliding clamp complex critical to DNA replication and repair, on a specific lysine residue (K164) is a key step in the activation of the DNA damage tolerance pathways. PCNA forms a homotrimeric ring encircling DNA, binding and coordinating the activities of a broad range of proteins, including DNA polymerases and many replication, repair, and regulatory factors (reviewed in refs. [7–11]). Monoubiquitination of PCNA triggers the TLS pathway, whereas further polyubiquitination of this ubiquitin moiety (linked to ubiquitin’s K63 residue, rather than the K48 involved in proteasomal degradation) involves additional factors and initiates the alternative pathway of template switching. Once loaded onto DNA, an ATP-dependent process facilitated by replication factor C (RFC), PCNA becomes capable of being monoubiquitinated on K164 by the E2 ubiquitin-conjugating enzyme Rad6 in complex with the E3 ubiquitin ligase Rad18, the former being the catalyst and the latter the adaptor for specific substrate recognition. The ubiquitination reaction cascade begins with the activation of the carboxy terminus of ubiquitin, catalyzed by the E1 ubiquitin-activating enzyme Uba1 (also known as UBE1), through reaction with ATP to form a ubiquitin adenylate intermediate, with subsequent steps not requiring ATP. The ubiquitin adenylate, still bound to Uba1, then reacts with a catalytic cysteine residue on Uba1, resulting in a Uba1~ubiquitin thioester conjugate. The ubiquitin is subsequently transferred to Rad6 to generate a Rad6~ubiquitin thioester conjugate. In complex with Rad18, the thioester then reacts with the side-chain amine of K164 on PCNA to form a more stable PCNA–ubiquitin isopeptide bond. The Rad6–Rad18 dimer can also autoubiquitinate Rad18 at multiple sites in vitro and in the cell, which appears to affect its function, subcellular localization, and stability [12, 13].

The human genome encodes two ubiquitin-specific E1 enzymes, approximately 40 ubiquitin-specific E2 enzymes, and over 600 ubiquitin-specific E3 proteins, with pathway selectivity narrowing as the sequence proceeds, ultimately involving recognition of a given substrate by a specific E3 protein. The vast majority of E3 ubiquitin ligases, such as Rad18, are of the so-called RING class, functioning as adaptors between a given E2 protein and a specific substrate protein, with transfer of ubiquitin from the E2 protein to the ultimate substrate. There are also two other types of E3 proteins, the HECT and RING-Between-RING classes, that themselves also form thioester conjugates with ubiquitin, as a third and final level of thioester-forming enzyme to mediate transfer of ubiquitin to the ultimate substrate (reviewed in refs. [14–17]). In addition to ubiquitin-specific E1, E2, and E3 proteins, there are cognate UBL-specific proteins, with some functional overlap between them. There are, therefore, a huge number of potential pathways to probe.

The high-throughput assays we report here to probe the PCNA ubiquitination cascade at different levels are all reconstituted systems, composed of the purified proteins relevant to each specific assay, adapted to the amplified luminescent proximity homogeneous assay (Alpha) system. The Alpha system has various advantages over other proximity-based approaches, such as the enzyme-linked immunosorbent assay (ELISA) and resonance energy transfer (RET) methods (e.g. fluorescence resonance energy transfer and bioluminescence resonance energy transfer) in terms of flexibility, adaptability, and sensitivity (reviewed in ref. [18]). Unlike conventional ELISAs, the Alpha system is a solution-phase and homogeneous assay—not requiring wash and separation steps—and has greater sensitivity and wider dynamic range. In comparison to RET methods, an advantage of the Alpha system is that it works without the stringent short distance and orientation restrictions on the donor–acceptor pair. Tagging or labeling two proteins (or other molecules) with any of the many tags or labels recognized by available Alpha bead coatings is generally enough to develop a workable Alpha system, without the laborious trial-and-error of developing a suitably coupled RET pair system.

We found the high-throughput Alpha assays for the different steps in the PCNA ubiquitination cascade to be robust, reliable, and sensitive. We found half-maximal inhibitory concentration (IC_50_) values and structure–activity relationship trends for Uba1 inhibitors we previously reported [19] as determined by the Alpha assay generally corresponding to other more traditional methods in terms of both inhibition of the overall PCNA ubiquitination cascade and Uba1 specifically. Thus, the system performs well as a screening tool for this reaction sequence and as a legitimate quantitative method for measuring ubiquitination and formation of thioester conjugates. The approaches should be straightforwardly adaptable to other ubiquitination or UBL post-translational modification cascades.

## Results

### Overview of study

**Fig. 1:**
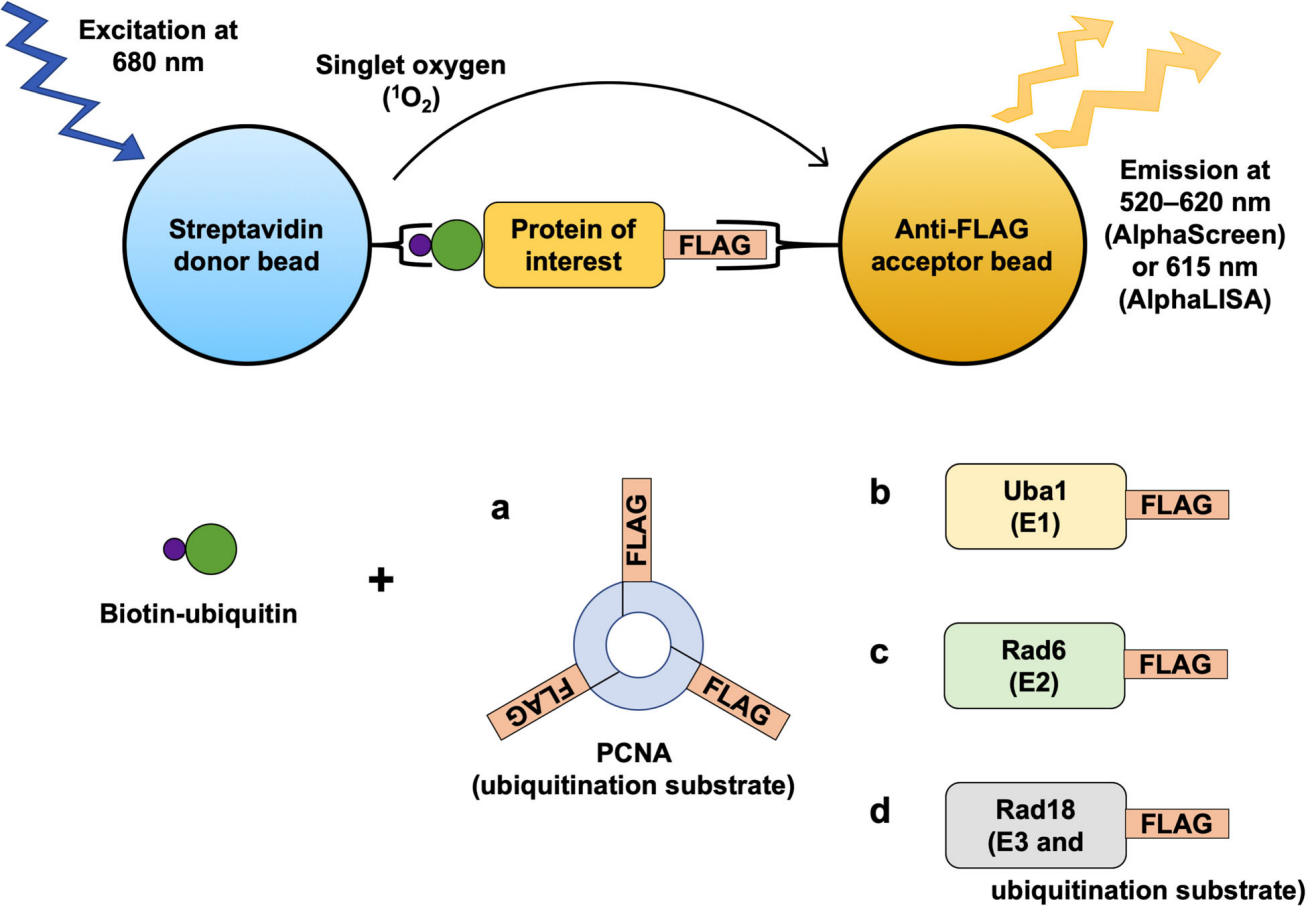
Diagram of the Alpha assays for: **a** PCNA ubiquitination, **b** Uba1~ubiquitin thioester formation, **c** Rad6~ubiquitin thioester formation, and **d** Rad18 autoubiquitination

Starting from conditions we optimized previously for Western blot analysis and quantitation of PCNA ubiquitination, as well as gel-based analyses of the formation of Uba1~ubiquitin thioester and Rad6~ubiquitin thioester conjugates [19], we further refined the reconstituted systems for the more sensitive Alpha technology. We sought to reduce the concentrations of the components in the reactions and also optimize the subsequent detection step of the assays. In the process, we developed a high-throughput Alpha-based PCNA ubiquitination assay that balances high sensitivity with low consumption of reagents, as well as analogous Alpha assays to probe Uba1~ubiquitin thioester formation, Rad6~ubiquitin thioester formation, and Rad18 autoubiquitination (Fig. 1).

### Optimization of the Alpha assay for PCNA ubiquitination

**Fig. 2:**
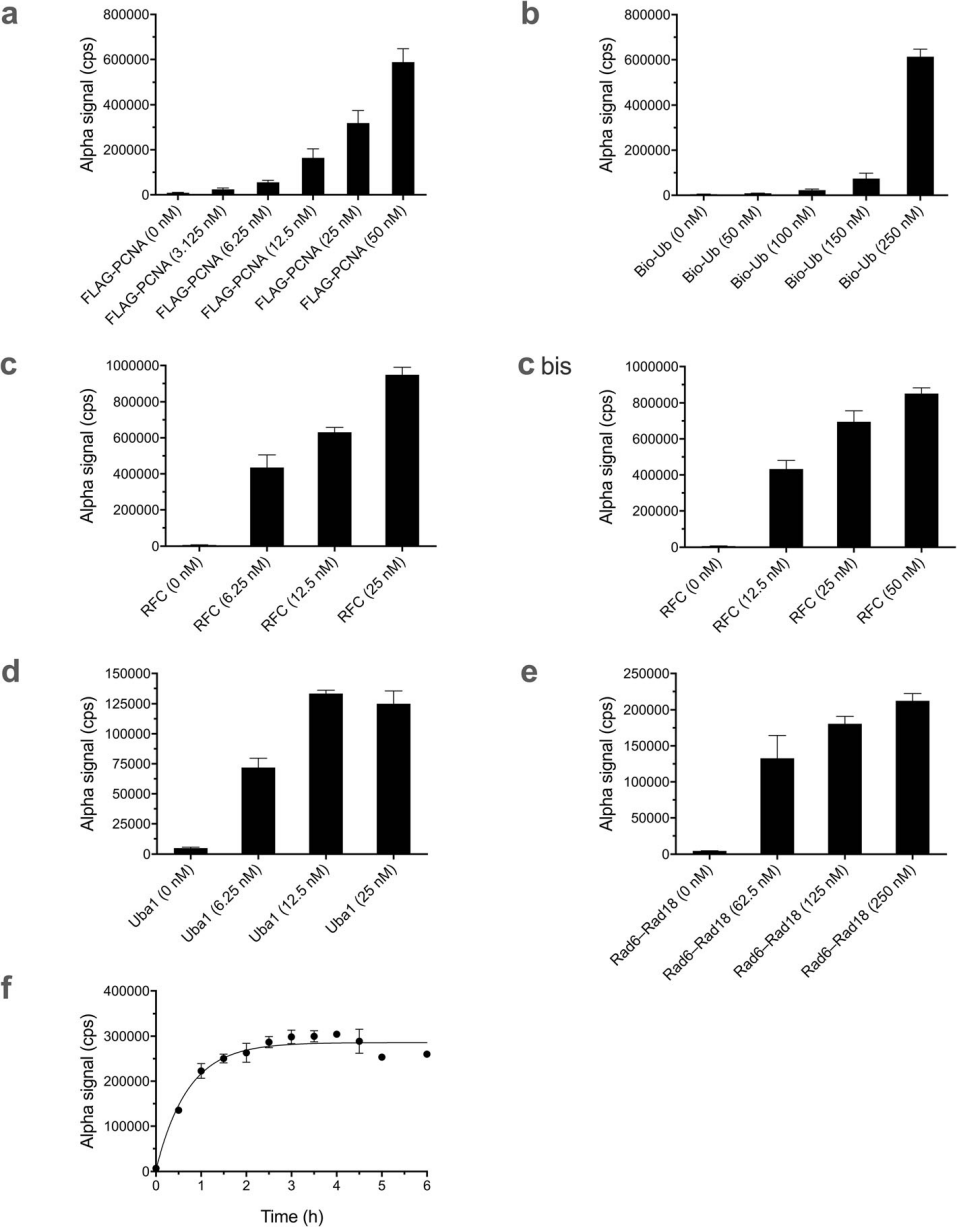
Alpha assay for PCNA ubiquitination. Data represent mean and standard deviation (SD) for 3–6 replicate samples in each case. Variable concentrations of: **a** FLAG-PCNA, **b** biotin-ubiquitin (Bio-Ub), **c** RFC (left and right panels are different concentration ranges from separate experiments), **d** Uba1, and **e** Rad6–Rad18 dimer, with other components in each case held constant as noted in Methods. **f** Reaction kinetics under final optimized conditions. Details are described in Methods

As a departure point, we began with the conditions we had found provided virtually complete ubiquitination of PCNA readily detectable by Western blot analysis [19]. We individually varied concentrations of FLAG-PCNA (Fig. 2a), biotin-ubiquitin (Fig. 2b), RFC (Fig. 2c), Uba1 (Fig. 2d), and Rad6–Rad18 dimer (Fig. 2e). In each case, we ascertained optimal concentration ranges for strong Alpha signals.

To investigate miniaturization questions, we evaluated the reaction done in different volumes (Additional file 1: Fig. S1). We also investigated the optimal parameters for Alpha detection after the PCNA ubiquitination reaction, looking at different dilution factors (Additional file 2: Fig. S2), donor and acceptor bead concentrations (Additional file 3: Fig. S3a), donor and acceptor bead ratios (Additional file 3: Fig. S3b), order of addition of donor and acceptor beads (Additional file 4: Fig. S4), and bead incubation times prior to detection (Additional file 5: Fig. S5). We found best results with 10× dilution and simultaneous addition of donor and acceptor beads. In terms of signal, while 20 μg/ml yielded stronger signals, we decided on 10 μg/ml to reduce bead consumption, since it gave good results nonetheless. With regard to incubation time with beads, longer incubations resulted in better signals.

Based on all the preceding experiments on the PCNA ubiquitination reaction and the Alpha detection step of the overall assay, we chose final concentrations, conditions, and procedures that balanced sensitivity and assay quality with the practical concerns of usage of proteins and beads, as described in Methods. Under these adopted conditions, we conducted a kinetics experiment to establish the time course of the reaction, with saturation reached around 2 h at 25 °C (Fig. 2f).

With protein concentrations for the PCNA ubiquitination cascade and Alpha conditions set, we investigated the dependence of the PCNA ubiquitination cascade on ATP concentration, which appeared to become non-limiting for an incubation of 2 h at 25 °C in the high micromolar range (Additional file 6: Fig. S6a). We also probed dimethyl sulfoxide (DMSO) tolerance of the reaction, as DMSO is the most commonly used carrier solvent for compound delivery in screening (Additional file 6: Fig. S6b).

We found Z′ factors had typical values for any given plate of between 0.6 and 0.9, particularly by robust statistics, which is less sensitive to outliers than standard statistics, when comparing positive (with ATP) and negative (without ATP) control values on each plate, indicating suitability for high-throughput screening. Strictly standardized mean difference values, signal-to-noise ratios, signal-to-background ratios, and signal window values per plate were generally also very good.

### Alpha assays for Uba1~ubiquitin, Rad6~ubiquitin, and Rad18 autoubiquitination

**Fig. 3:**
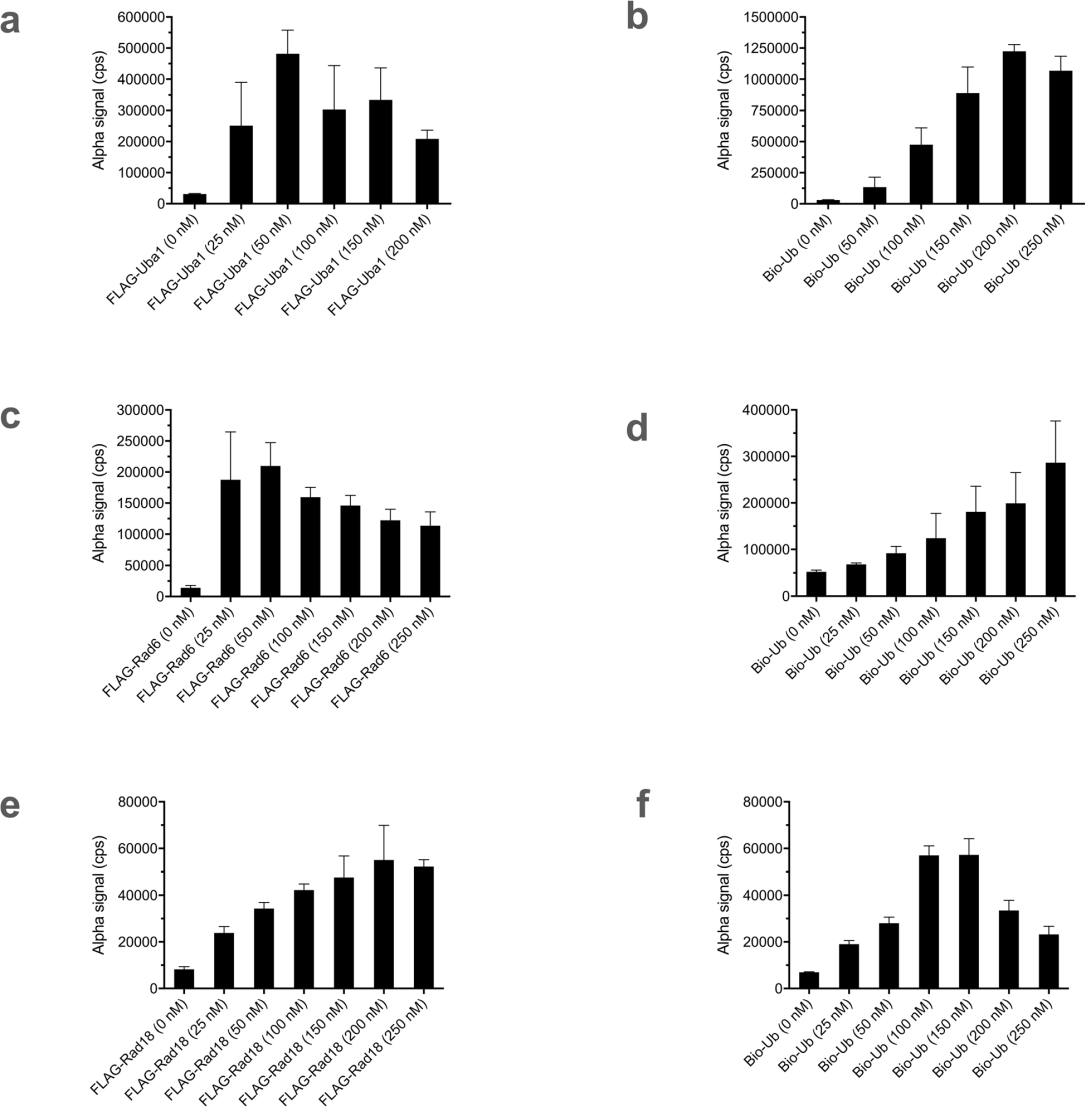
Alpha assays for Uba1~ubiquitin thioester formation, Rad6~ubiquitin thioester formation, and Rad18 ubiquitination. Data represent mean and SD for 5 samples in each case. Variable concentrations of **a** FLAG-Uba1 and **b** biotin-ubiquitin (Bio-Ub) in the Uba1~ubiquitin thioester formation assay, with the other component in each case being held constant. Variable concentrations of **c** FLAG-Rad6 and **d** biotin-ubiquitin in the Rad6~ubiquitin thioester formation assay. Variable concentrations of **e** FLAG-Rad18 and **f** biotin-ubiquitin in the Rad18 autoubiquitination assay. Details for each assay are described in Methods

We developed three additional Alpha assays to probe other steps in the PCNA ubiquitination cascade. First, we adapted the Alpha system for quantitative evaluation of Uba1~ubiquitin thioester formation, again starting with the conditions we previously worked out for gel-based detection [19] and then refined for Alpha detection. We used a FLAG-Uba1 construct and individually varied concentrations of the analytes, FLAG-Uba1 (Fig. 3a), with the decline in signal at higher concentrations the probable result of the hook effect (see Discussion), and biotin-ubiquitin (Fig. 3b), finding conditions yielding good results.

Second, we developed an Alpha assay for detection of the Rad6~ubiquitin thioester conjugate, again starting from conditions previously developed for gel-based detection [19]. We made a FLAG-Rad6 construct and performed reactions with non-FLAG-tagged Uba1 and biotin-ubiquitin. We individually varied concentrations of Rad6 (Fig. 3c) and biotin-ubiquitin (Fig. 3d), again defining conditions that gave satisfactory results.

We then split the two-reaction sequence into two steps to allow for more narrow screening for direct Rad6 modulators as distinct from those that secondarily affect Rad6 by instead directly targeting Uba1, with a first step for Uba1 charging with ubiquitin and then a second step where the precharged Uba1~ubiquitin was combined with Rad6 (preincubated with compound in a screening setting) for the transthioesterification reaction (Additional file 7: Fig. S7a). We experimented with adding ethylenediaminetetraacetic acid (EDTA) to the charged Uba1~ubiquitin samples before adding Rad6 to chelate the Mg^2+^ and prevent further ATP-dependent charging of Uba1 with ubiquitin after being mixed with Rad6 so that the assay would be potentially a yet cleaner transthioesterification readout to evaluate Rad6~ubiquitin thioester formation solely. This would mitigate the possibility of picking up false positives from the Rad6~ubiquitin thioester formation assay that bleed over and inhibit Uba1 instead. At a final EDTA concentration of 20 mM, the same concentration employed above to terminate the overall PCNA ubiquitination reaction sequence before the Alpha step, we found diminished transthioesterification from Uba1 to Rad6 for some reason, despite being a reaction not requiring Mg^2+^-ATP (Additional file 7: Fig. S7a). We thus reconfigured the Rad6~ubiquitin thioester formation assay with less ATP (250 μM) and MgCl_2_ (500 μM), then varied the concentration of EDTA for quenching Uba1 precharging, with little-to-no effect on the reaction up to 2 mM EDTA (Additional file 7: Fig. S7b). Another option for preventing further Uba1 charging would be adding apyrase to hydrolyze ATP between the steps.

Finally, we developed an Alpha assay for the detection of Rad18 autoubiquitination. We constructed a FLAG-Rad18 construct and performed reactions with non-FLAG-tagged Uba1, Rad6–Rad18 dimer, and biotin-ubiquitin. Variation of concentrations of FLAG-Rad18 (Fig. 3e) and biotin-ubiquitin (Fig. 3f) for this reaction was also performed, and we again found conditions that resulted in good outcomes. We then once more implemented a quench of Uba1 charging with EDTA, as we did with the Rad6~ubiquitin thioester formation reaction in a split assay (Additional file 7: Fig. S7c), again allowing for elimination of any bleedover effects of compound on Uba1.

**Fig. 4:**
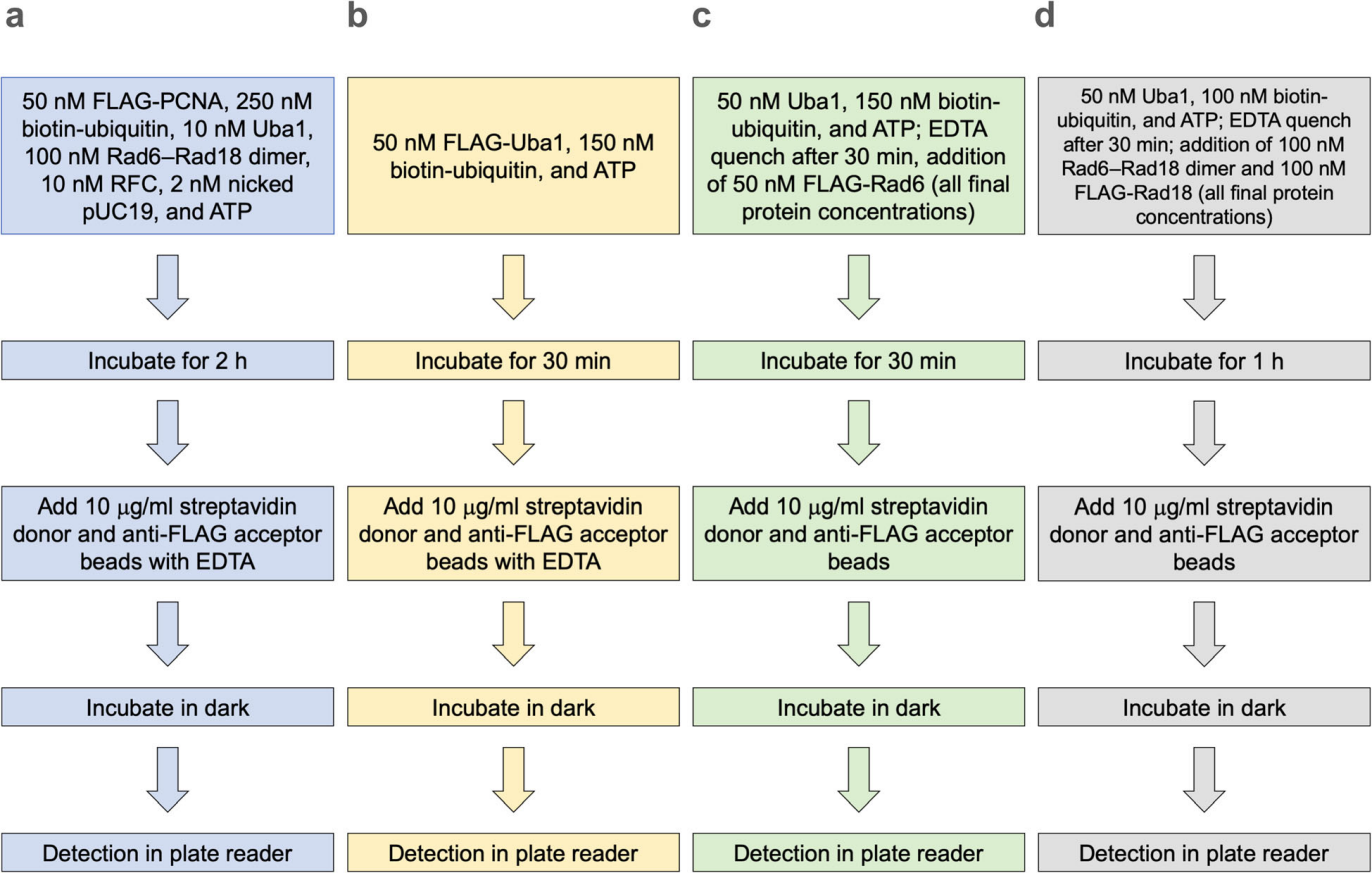
Flowchart of optimized conditions for each Alpha assay. Stepwise procedures are shown for: **a** PCNA ubiquitination, **b** Uba1~ubiquitin thioester formation, **c** Rad6~ubiquitin thioester formation, **d** Rad18 autoubiquitination. Detailed conditions are described in Methods

In choosing conditions for each assay to use for screening, we balanced the desire to reduce protein concentrations with retaining good signals. Flowcharts for the conditions and procedures for the four Alpha assays (PCNA ubiquitination, Uba1~ubiquitin thioester formation, Rad6~ubiquitin thioester formation, and Rad18 autoubiquitination) are shown in Fig. 4.

### Evaluation of the performance of the Alpha assays for PCNA ubiquitination and Uba1~ubiquitin thioester formation by testing with already identified inhibitors

**Fig. 5:**
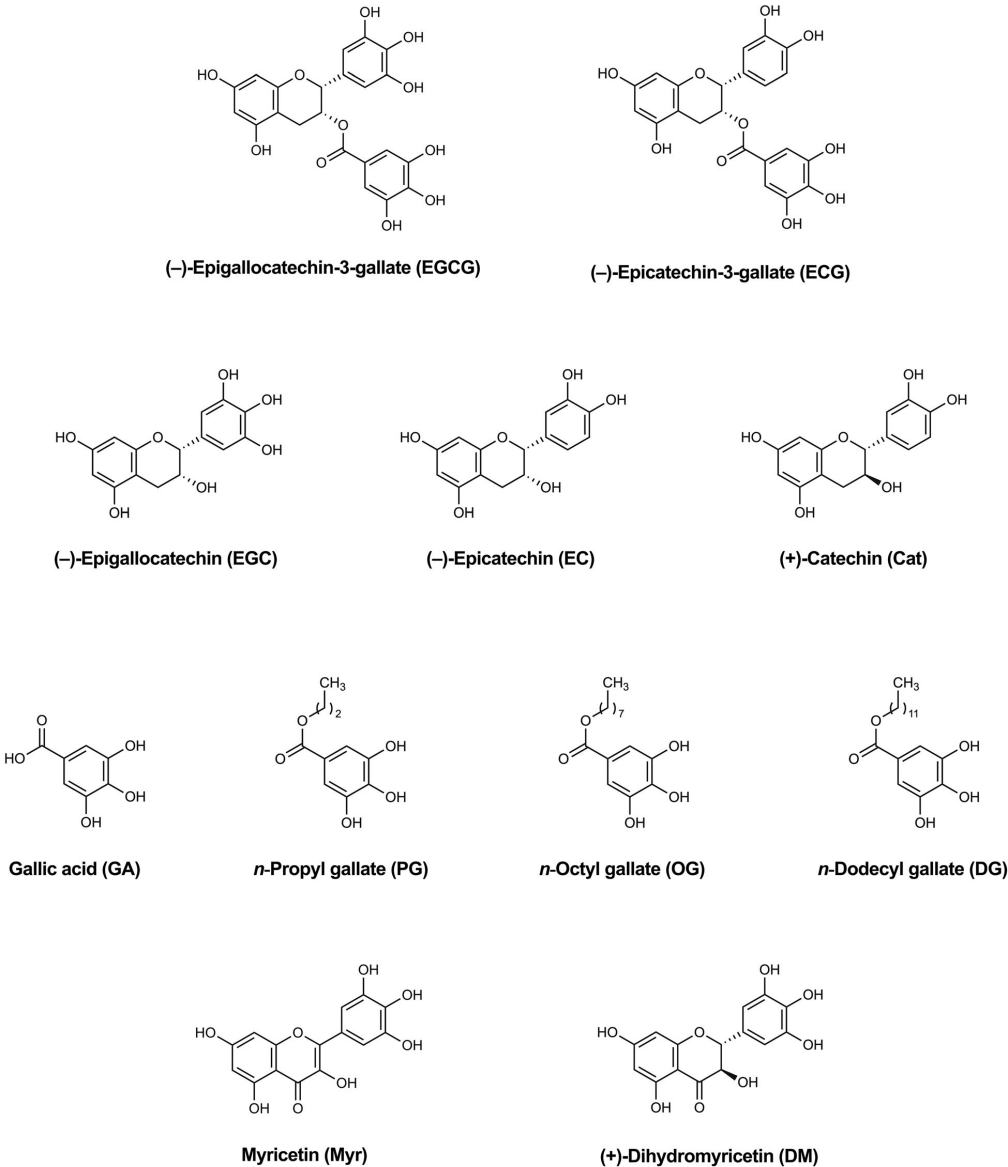
Structures of Uba1 inhibitors and their analogs examined in the Alpha assays. The compounds, some of which inhibit Uba1 and ubiquitination, as we previously reported [19]**,** were: (−)-epigallocatechin-3-gallate (EGCG), (−)-epicatechin-3-gallate (ECG), (−)-epigallocatechin (EGC), (−)-epicatechin (EC), (+)-catechin (Cat), gallic acid (GA), *n*-propyl gallate (PG), *n*-octyl gallate (OG), *n*-dodecyl (lauryl) gallate (DG), myricetin (Myr), and (+)-dihydromyricetin (DM)

**Fig. 6:**
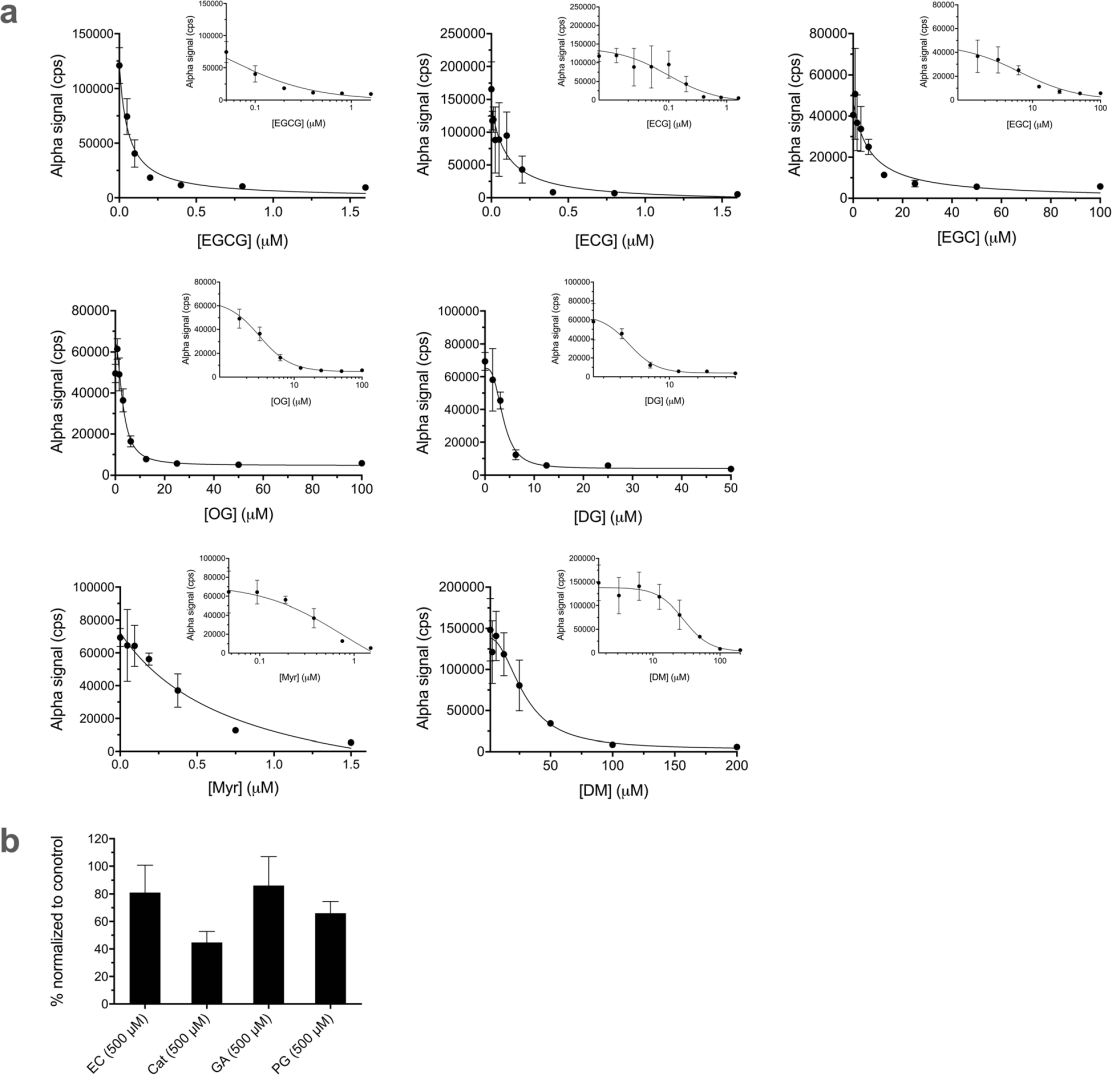
Effects of compounds in the PCNA ubiquitination Alpha assay. Samples were preincubated with compounds for 15 min prior to initiation of reactions with ATP. Data represent mean and SD for 3–7 samples in each case. Final DMSO concentration was 1% in all cases. **a** Dose–response for bioactive inhibitors of PCNA ubiquitination, plotted both linearly and semi-logarithmically (inset). **b** Effects of compounds with little activity against PCNA ubiquitination at 500 μM, normalized to positive (DMSO alone) control values

**Fig. 7:**
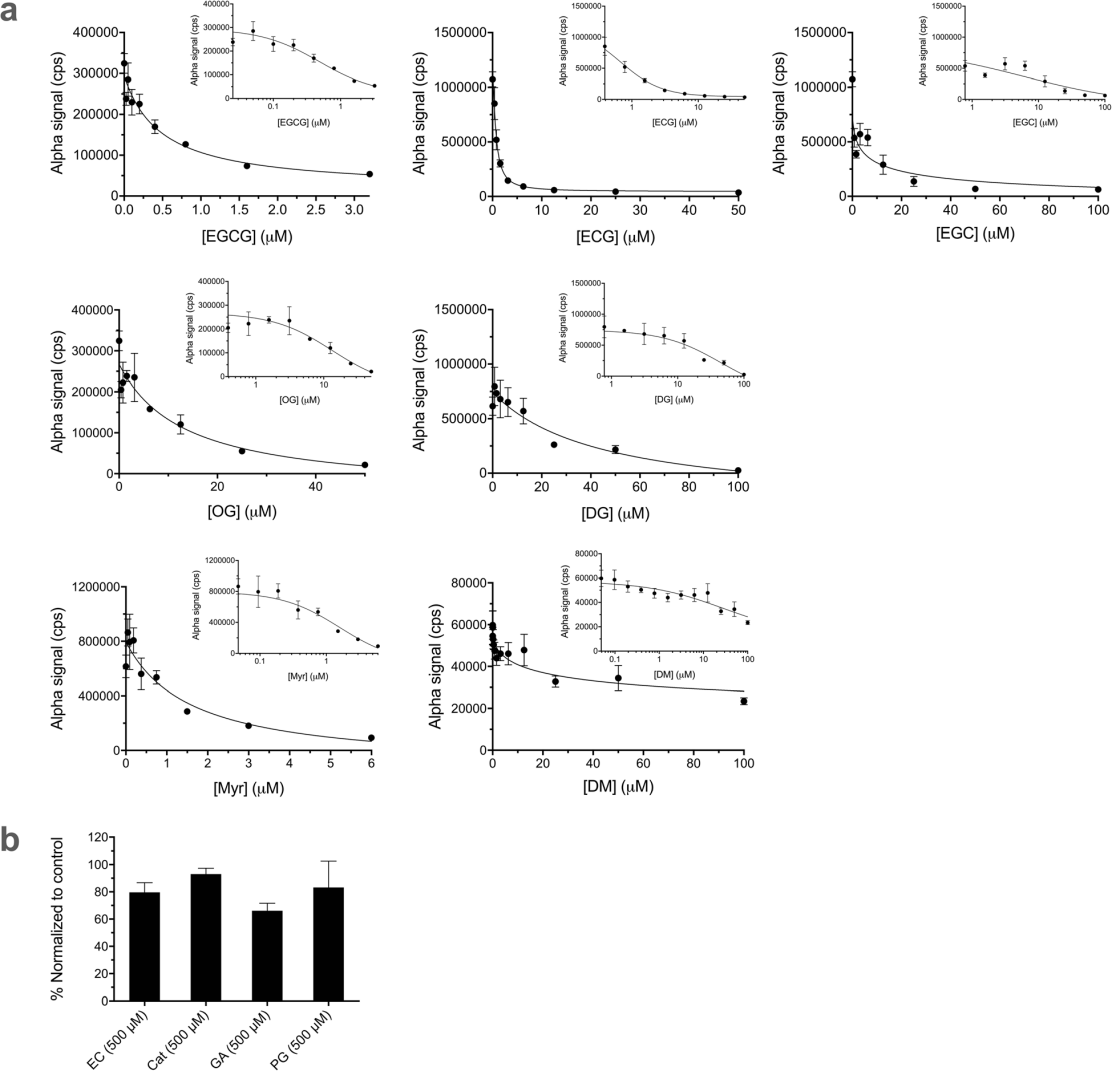
Effects of compounds in the Uba1~ubiquitin thioester formation Alpha assay. Data represent mean and SD for 3–5 samples in each case. Final DMSO concentration was 1% in all cases. FLAG-Uba1 samples were preincubated with compounds for 15 min prior to addition of biotin-ubiquitin and ATP. **a** Dose–response for bioactive inhibitors of Uba1~ubiquitin thioester formation, plotted both linearly and semi-logarithmically (inset). **b** Effects of compounds with little activity against Uba1~ubiquitin thioester formation at 500 μM, normalized to positive (DMSO alone) control values. Calculated IC_50_ values for the compounds are shown in Table 1

In addition to evaluating plate quality control metrics, we further validated the assays with inhibitors of Uba1 we recently discovered [19], a number of different green tea compounds (structures in Fig. 5). We performed dose–response experiments with the Alpha assays for PCNA ubiquitination (Fig. 6) and Uba1~ubiquitin thioester formation (Fig. 7a and b). The structure–activity relationships were similar to those we previously reported [19], with generally similar trends in relative calculated IC_50_ values for each of the bioactive compounds, as shown in Table 1.

**Table 1:** Half-maximal inhibitory concentration (IC_50_) values for compounds against PCNA ubiquitination and Uba1~ubiquitin thioester formation by the Alpha assay compared to other methods. All IC_50_, standard error (SE), and 95% confidence interval (CI) values listed were calculated by nonlinear regression with GraphPad Prism software. Ind. = indeterminate

**Compound**	**IC**_**50**_**for inhibition of PCNA ubiquitination (Alpha)**	**IC**_**50**_**for inhibition of PCNA ubiquitination** **(Western blot)** [19]	**IC**_**50**_**for inhibition of Uba1~Ub thioester formation** **(Alpha)**	**IC**_**50**_**for inhibition of Uba1~Ub thioester formation (gel-based)** [19]
EGCG	0.0566 μM (SE = 0.00954; CI: 0.0409–0.0776)	0.228 μM (SE = 0.0342; CI: 0.171–0.319)	0.488 μM (SE = 0.145; CI: 0.259–0.949)	1.63 μM (SE = 0.458; CI: 0.835–3.45)
ECG	0.0988 μM (SE = 0.0442; CI: 0.0318–0.275)	0.537 μM (SE = 0.0946; CI: 0.375–0.843)	0.769 μM (SE = 0.113; CI: 0.586–1.02)	4.22 μM (SE = 2.16; CI: 1.26–13.78)
EGC	6.62 μM (SE = 3.23; CI: 2.92–16.5)	43.3 μM (SE = 22.8; CI: 16.1–129)	5.96 μM (SE = 17.3; CI: 0.101–Ind.)	7.58 μM (SE = 3.87; CI: 2.93–20.7)
EC	> 500 μM	> 500 μM	> 500 μM	> 500 μM
Cat	ca. 500 μM	> 500 μM	> 500 μM	> 500 μM
GA	> 500 μM	> 500 μM	> 500 μM	> 500 μM
PG	> 500 μM	> 500 μM	> 500 μM	> 500 μM
OG	3.08 μM (SE = 0.611; CI: 2.59–6.35)	10.3 μM (SE = 6.41; CI: 3.18–40.1)	13.4 μM (SE = 6.02; CI: 5.34–38.5)	80.6 μM (SE = 33.9; CI: 34.8–405)
DG	3.73 μM (SE = 0.566; CI: 2.83–4.56)	1.63 μM (SE = 0.312; CI: 1.05–2.67)	44.4 μM (SE = 22.4; CI: 18.8–150)	35.0 μM (SE = 25.1; CI: 6.29–533)
Myr	0.760 μM (SE = 0.393; CI: 0.323–2.61)	1.21 μM (SE = 0.208; CI: 0.876–4.20)	1.59 μM (SE = 0.746; CI: 0.721–4.22)	0.721 μM (SE = 0.497; CI: 0.177–2.68)
DM	28.7 μM (SE = 5.30; CI: 19.7–49.5)	66.4 μM (SE = 38.9; CI: 27.2–181)	27**.**5 μM (SE = 13.6; CI: 7.52–113)	14.3 μM (SE = 3.14; CI: 5.93–22.2)

## Discussion

The PerkinElmer Alpha system is based on the luminescent oxygen channeling immunoassay, a homogeneous bead-based immunoassay method. High-energy irradiation at 680 nm excites the photosensitizer phthalocyanine in the Alpha donor beads, which converts ambient ground-state oxygen to an excited singlet state (distinct from the far more reactive superoxide radical), which has a half-life of ~ 4 μs in aqueous solutions and can diffuse over a distance of up to ~ 200 nm. If an Alpha acceptor bead is within this distance, the singlet oxygen can react with a thioxene derivative in the acceptor bead, producing a chemiluminescent emission that excites fluors also contained in the acceptor beads (anthracene and rubrene for the version of the assay known as AlphaScreen or a europium chelate for that known as AlphaLISA). The resulting fluorescent emissions (520–620 nm or 615 nm for AlphaScreen or AlphaLISA, respectively) are detected by a photomultiplier tube. AlphaLISA acceptor beads, with a more narrowly defined and sharp emission spectrum, are useful in certain samples, such as serum or plasma, where there may be components that interfere with the AlphaScreen emission. In the present study, we employed the AlphaScreen acceptor beads, but we have also worked with AlphaLISA acceptor beads in the PCNA ubiquitination assay and found no difference in performance between the two versions of the Alpha assay in our system [19].

We have applied the Alpha system to the PCNA ubiquitination cascade, first to the overall process and then to the different intermediary steps in the reaction sequence involving covalent ubiquitin adducts. Modulators of the different specific components of the PCNA ubiquitination cascade can be identified and characterized in terms of degree of potency readily, allowing for rapid discovery of both potential research probes and therapeutic agents with a broad spectrum of anticancer activity.

In addition to its utility as a high-throughput screening method with advantages over RET and ELISA approaches (reviewed in ref. [18]), the Alpha system is considerably quicker, simpler, and more sensitive than Western blot analysis and other traditional methods for low-throughput detection of ubiquitination or ubiquitin thioester conjugates. We found reasonably good correspondence in compound potency trends between the Alpha assay and traditional assays (Table 1) with the Uba1 inhibitors we previously reported [19]. In addition, the Alpha system has been used to probe components of other ubiquitination or UBL post-translational modification pathways [20–25]. Here we have adapted it to look at new ultimate substrate modifications (PCNA and Rad18) and also to evaluate intermediate ubiquitin conjugates in the pathway. The approach can be employed to screen not only for inhibitors but also for activators/enhancers of these processes, particularly if the screening is done at reaction times prior to normal attainment of saturating modification of a substrate.

Optimization of protein and bead concentrations and other parameters is important in the implementation of an Alpha-based screening effort from the standpoints of utilizing economical amounts of proteins and beads while retaining good signal strength and dynamic range. The overall Alpha assay can be viewed as composed of two main parts: first, the biochemical process of interest that generates the proximity relationship to be assayed; second, the procedures for Alpha detection (which involves dilution of the initial samples with addition of beads and then the actual detection itself). Both of these major steps of the overall assay must be optimized. We found that while the dynamic signal range was consistently good, graded, and reproducible, precise signal intensity values of the range (e.g. between minimum and maximum signal values or between negative and positive control values) can vary from experiment to experiment, even under ostensibly identical conditions; thus, combining data from multiple independent experiments should be done only after meaningful normalization. Another point to note is that the Alpha assay is susceptible to the high-dose hook effect—wherein too high concentrations of analytes can result in diminished signals in bimolecular detection assays involving saturable reagent binding such as in AlphaScreen/AlphaLISA, ELISA, and indirect-detection (e.g. antibody-based) RET systems—which plays into these considerations. (The hook effect, also referred to as the hooking or prozone effect, is named after the shape of the analyte concentration–signal curve in such cases, which resembles a fish hook.) Optimal concentrations, conditions, and procedures cannot be predicted a priori for any given experimental system. They will depend on factors such as the nature of the analytes, the analyte-capturing coating on the beads, their affinities, and the stoichiometries of their interactions. Optimal assay parameters must therefore be determined empirically for any given system.

## Conclusions

We have developed and validated multiple assays, based on the powerful Alpha technology, to assess ubiquitination of a substrate protein and the formation of ubiquitin thioester conjugates involved in the reaction cascade. We show that PCNA ubiquitination and the intermediate steps leading to ultimate substrate modification can be interrogated in a high-throughput manner that also directly yields externally verifiable results for valid measures of the potency of inhibitors for discrete steps in the process. The assays are straightforward to implement, reliable, robust, and quantitative. The approach can be readily adapted to addressing both overall and step-specific events in other ubiquitination and UBL post-translational modification cascades.

## Methods

### Proteins and reagents

All proteins were of human origin, except for RFC, which was from *Saccharomyces cerevisiae*, and were expressed and purified as previously described [19, 26–29], as detailed below. A sodium dodecyl sulfate (SDS)–polyacrylamide gel of representative protein preparations is shown in Additional file 8: Fig. S8. Biotinylated human ubiquitin was purchased from Boston Biochem/R&D Systems (UB-570). Purified pUC19 plasmid was nicked with Nt.BstNBI (New England Biolabs) at 55 °C overnight. Streptavidin donor and anti-FLAG acceptor AlphaScreen beads were from PerkinElmer.

### His-tagged Uba1 preparation

His-tagged human Uba1 in the pET3a bacterial expression vector (Addgene plasmid #63571, courtesy of Titia Sixma) was expressed in *Escherichia coli* strain BL21-CodonPlus (DE3)-RIL (Agilent). The cells were centrifuged and washed in 1× phosphate-buffered saline (PBS). The cell suspensions were dropped into liquid nitrogen, and the resulting frozen beads were ground with a SPEX SamplePrep 6775 Freezer/Mill. The lysates were centrifuged, and the supernatant was applied to a Ni-nitrilotriacetic acid (NTA) agarose (Machery-Nagel) column. Following repeated washings, the protein was eluted from the column with 250 mM imidazole, then dialyzed against 20 mM Tris-HCl, pH 7.5, 150 mM NaCl, 10% glycerol, 0.01% NP-40, and 1 mM freshly added dithiothreitol (DTT).

### Rad6–Rad18 dimer preparation

Both human Rad6B and glutathione *S*-transferase (GST)-fused human Rad18 constructs (each cloned into the pBJ842 yeast expression vector, which contains Leu and Trp auxotrophic markers) were introduced into the *S. cerevisiae* BJ5654 strain. The cells were grown in omission media (−Leu, −Trp), then collected when reaching an OD_600_ of 0.8–1, centrifuged, and washed with 1× PBS. The cells were resuspended in yeast lysis buffer consisting of 50 mM HEPES, pH 7.5, 50 mM KCl, 300 mM NaCl, 10% sucrose, 0.5 mM EDTA, and 2.8 mM β-mercaptoethanol). The cell lysate was then dropped into liquid nitrogen, then ground with a SPEX SamplePrep 6775 Freezer/Mill, collected into 1.5 ml microcentrifuge tubes, and centrifuged. The supernatant was applied to a glutathione Sepharose 4B (GE Healthcare) column, followed by repeated washing with 20 mM Tris-HCl, pH 7.5, 10% glycerol, 0.01% NP-40, and 1 mM DTT at progressively lower NaCl concentrations (3× 500 mM, 3× 250 mM, 1× 150 mM). The GST moiety was cleaved with PreScission Protease (GE Healthcare) with incubation for 2 h at 4 °C lightly on a shaker, and the dimer was eluted with a 1.5× bed volume equivalent of 20 mM Tris-HCl, pH 7.5, 150 mM NaCl, 10% glycerol, 0.01% NP-40, and 1 mM DTT.

### FLAG-tagged PCNA and FLAG-tagged Uba1 preparation

Constructs of human PCNA bearing GST and FLAG tags or human Uba1 bearing GST and FLAG tags, both cloned into the pBJ842 yeast expression vector, were used to prepare FLAG-tagged PCNA and FLAG-tagged Uba1 proteins, respectively, with subsequent expression in the *S. cerevisiae* BJ5654 strain and purification with cleavage of the GST moiety, as above.

### FLAG-tagged Rad6 and FLAG-tagged Rad18 preparation

Constructs were generated with Gateway cloning technology (Life Technologies). Human Rad6B and human Rad18 cDNA sequences from entry constructs were recombined into a modified pGEX-6P-1 (Amersham) destination vector bearing GST and FLAG tags with a Gateway cassette via the LR Clonase II reaction (Invitrogen). Proteins were overexpressed in the *E. coli* strain BL21-CodonPlus (DE3)-RIL (Agilent). The proteins were purified with removal of the GST moiety, as above.

### RFC preparation

The construct pLANT-2/RIL–RFC[1^s^ + *5*] was cotransformed with the construct pET(11a)–RFC[2 + *3 + 4*] [26] into the *E. coli* strain BL21-CodonPlus (DE3)-RIL (Agilent), where RFC1^s^ represents an N-terminally truncated form of the large RFC subunit [30]. The cells were plated and allowed to grow under selection with ampicillin (100 μg/ml) and kanamycin (50 μg/ml) overnight. A single transformant colony was then picked and grown in 2 ml of Luria-Bertani medium containing ampicillin (100 μg/ml) and kanamycin (50 μg/ml) at 37 °C for 8 h, then inoculated into a started culture of 2 l of Luria-Bertani medium containing ampicillin (100 μg/ml) and kanamycin (50 μg/ml) for 16 h. 300 ml of the starter culture was inoculated into 2 l of Luria-Bertani medium and grown to an OD_600_ of 0.8 at 37 °C. The cultures were cooled down to 16 °C and induced with 0.5 mM isopropyl-β-d-thiogalactoside for 16 h. All further steps were performed at 4 °C.

The cells were harvested by centrifugation and then resuspended in HEG buffer (30 mM HEPES, pH 7.6, 0.5 mM EDTA, 10% glycerol, 5 mM β-mercaptoethanol) containing 150 mM NaCl. To lyse the cells, lysozyme was added to 0.4 mg/ml, and the cells were subjected to three freeze-thaw cycles, followed by mechanical shearing through a hypodermic needle. The cell lysate was treated with Benzonase endonuclease, purity grade II (Merck), according to the manufacturer’s protocol. The cell lysate was clarified by centrifugation. RFC was purified by chromatography over an SP Sepharose column (bed volume of 6 ml), pre-equilibrated with HEG containing 50 mM NaCl, followed by wash with 60 ml of HEG buffer containing 50 mM NaCl. Elution was carried with a gradient of 50–1000 mM NaCl in 60 ml of HEG buffer. Peak fractions were collected, pooled, then diluted with Ni-NTA buffer (30 mM HEPES, pH 7.6, 20 mM imidazole, 500 mM NaCl, 10% glycerol, 5 mM β-mercaptoethanol). The resulting sample was then applied to a Ni-NTA agarose (Machery-Nagel) column (bed volume of 500 μl), pre-equilibrated with Ni-NTA buffer. The column was then washed with 5 ml of Ni-NTA buffer, and proteins were eluted by a three-step gradient (100 mM, 250 mM, and 500 mM imidazole), each with 1.5 ml overall volume. Fractions were tested for PCNA loading ability, and peak fractions were aliquoted, frozen in liquid N_2_, and stored at − 80 °C until subsequent use.

### Alpha assay for PCNA ubiquitination

Reactions were performed in 96-well white round-bottom polypropylene plates (Greiner Bio-One) in a buffer consisting of 40 mM Tris-HCl, pH 7.5, 8 mM MgCl_2_, and 10% glycerol, with protein concentrations as noted below. Reactions were initiated with ATP added to a final concentration of 2 mM (unless otherwise noted), with incubation for 2 h at 25 °C (except for time-course experiments, where different incubation times were examined). The samples were then diluted in a buffer of 25 mM HEPES, pH 7.5, 100 mM NaCl, 0.1% Tween 20, and 1 mM DTT (Alpha buffer), containing 20 mM EDTA and streptavidin donor and anti-FLAG AlphaScreen acceptor beads, under low-light conditions with dark yellow-green filter (LEE 090) covering sources of lighting. (In the case of time-course experiments, however, the EDTA, which terminates both the ATP-dependent PCNA-loading process and the ubiquitination reaction cascade by chelating Mg^2+^, was instead added separately as a prior step until all samples were ready for Alpha detection.) In the case of the two-part assays involving quenching of Uba1 charging, EDTA was added to the Uba1 sample before the subsequent components and not with the donor and acceptor bead solution. Following incubation as indicated below, plates were read in a Tecan Spark plate reader (equipped with a dedicated high-energy laser light source and both cooling-and-heating temperature control for temperature consistency), with excitation at 680 nm and measurement of emission at 520–620 nm, as appropriate for the AlphaScreen acceptor beads.

### FLAG-PCNA concentration variation in the PCNA ubiquitination assay

Different FLAG-PCNA concentrations were tested with the other reaction components held constant at 2.5 nM nicked pUC19, 50 nM RFC, 50 nM Uba1, 250 nM Rad6–Rad18 dimer, and 250 nM biotin-ubiquitin. The reactions were initiated by addition of ATP to 2 mM for a final volume of 20 μl with incubation for 2 h at 25 °C. (Note: The above conditions, serving as a starting point, are those we had previously optimized for virtually complete ubiquitination of PCNA as detected by Western blot analysis [19]). For the Alpha assay itself, the samples were diluted by a factor of 10× in Alpha buffer with donor and acceptor beads (20 μg/ml each) and 20 mM EDTA. Following incubation with beads for 1 h at 25 °C, plates were read in the plate reader (Fig. 2a).

### Biotin-ubiquitin concentration variation in the PCNA ubiquitination assay

Different biotin-ubiquitin concentrations were tested with the other reaction components held constant at 2.5 nM nicked pUC19, 50 nM RFC, 50 nM Uba1, 250 nM Rad6–Rad18 dimer, and 50 nM FLAG-PCNA, with the assay otherwise carried out as above (Fig. 2b).

### RFC concentration variation in the PCNA ubiquitination assay

Different RFC concentrations were tested with the other reaction components held constant at 2.5 nM nicked pUC19, 50 nM Uba1, 250 nM Rad6–Rad18 dimer, 50 nM FLAG-PCNA, and 250 nM biotin-ubiquitin, with the assay carried out otherwise as above (Fig. 2c).

### Uba1 concentration variation in the PCNA ubiquitination assay

Different Uba1 concentrations were tested with the other reaction components held constant at 2.5 nM nicked pUC19, 50 nM RFC, 250 nM Rad6–Rad18 dimer, 50 nM FLAG-PCNA, and 250 nM biotin-ubiquitin, with the assay carried out otherwise as above (Fig. 2d).

### Rad6–Rad18 concentration variation in the PCNA ubiquitination assay

Different Rad6–Rad18 dimer concentrations were tested with the other reaction components held constant at 2.5 nM nicked pUC19, 50 nM RFC, 50 nM Uba1, 250 nM Rad6–Rad18 dimer, 50 nM FLAG-PCNA, and 250 nM biotin-ubiquitin, with the assay carried out otherwise as above (Fig. 2e).

### Optimized protein concentrations for the PCNA ubiquitination cascade

Based on the above experiments, we standardized on protein concentrations of 10 nM RFC (though below optimal, we wished to lessen usage of the most onerous component of the system to express and purify), 10 nM Uba1, 100 nM Rad6–Rad18 dimer, 50 nM FLAG-PCNA, and 250 nM biotin-ubiquitin for the reaction component of the assay. The experiments below were thus conducted with these concentrations.

### PCNA ubiquitination reaction volumes

Reactions were performed in different reaction volumes with incubation for 2 h at 25 °C, followed by Alpha detection after 10× dilution in Alpha buffer containing donor and acceptor beads (20 μg/ml each) and 20 mM EDTA, and incubation in the dark for 4 h at 25 °C prior to Alpha detection (Additional file 1: Fig. S1), as longer incubation time with beads resulted in improved signal (see Additional file 5: Fig. S5). Subsequent reactions were done in 20 μl, also with incubation in the dark for 4 h at 25 °C.

### Dilution factors for detection of ubiquitinated PCNA

Reactions were diluted to different degrees in Alpha buffer containing donor and acceptor beads (20 μg/ml each) and 20 mM EDTA, with the assay otherwise carried out as above (Additional file 2: Fig. S2).

### Donor and acceptor bead concentrations for detection of ubiquitinated PCNA

Reactions were diluted 10× in Alpha buffer with different equimolar donor and acceptor bead concentrations and 20 mM EDTA, with the assay otherwise carried out as above (Additional file 3: Fig. S3a).

### Donor and acceptor bead ratios for detection of ubiquitinated PCNA

Reactions were diluted 10× in Alpha buffer with different donor:acceptor bead concentration ratios and 20 mM EDTA, with the assay otherwise carried out as above (Additional file 3: Fig. S3b).

### Donor and acceptor bead order of addition for detection of ubiquitinated PCNA

Reactions were diluted in stepwise fashion in equal volumes (for final dilution of 10× in Alpha buffer with donor and acceptor beads at 10 μg/ml final concentration for each), as follows: (1) donor beads first (with incubation for 2 h), then acceptor beads (with further incubation for 2 h), or (2) acceptor beads first (with incubation for 2 h), then donor beads (with further incubation for 2 h), or (3) donor and acceptor beads added simultaneously (with incubation for 2 or 4 h), all at 25 °C, followed by incubation and Alpha detection (Additional file 4: Fig. S4).

### Donor and acceptor bead incubation times for detection of ubiquitinated PCNA

After reactions, samples were diluted by 10× in Alpha buffer containing donor and acceptor beads (10 μg/ml each) for different lengths of time (30 min to 12 h) before Alpha detection (Additional file 5: Fig. S5).

### Final optimized conditions for the PCNA ubiquitination cascade and Alpha detection

In the end, based on the sum of experimental results for conditions of both the PCNA ubiquitination reaction and the Alpha detection steps of the overall assay, the final conditions chosen to balance both optimal assaying and material usage concerns for the PCNA ubiquitination assay were: 2 nM nicked pUC19 (we reduced the nicked pUC19 concentration to an even 2 nM, as the precise DNA concentration makes little difference to the reaction), 10 nM RFC, 10 nM Uba1, 100 nM Rad6–Rad18 dimer, 50 nM FLAG-PCNA, and 250 nM biotin-ubiquitin; initiation with 2 mM ATP; a reaction volume of 20 μl in 96-well plates, with incubation for 2 h at 25 °C; dilution by a factor of 10× in Alpha buffer containing donor and acceptor beads (10 μg/ml each) and 20 mM EDTA, with incubation of 4 h in the dark at 25 °C, followed by Alpha detection. In 384-well plates (with a reaction volume of 10 μl, followed by 10× dilution and Alpha detection as above), the reaction also proceeded, but with less consistency.

### PCNA ubiquitination reaction kinetics under final optimized conditions

Reactions were stopped at different time intervals from 0 to 6 h by adding EDTA to 20 mM and saved until the Alpha detection step, at which point the samples were diluted 10× in Alpha buffer containing donor and acceptor beads (10 μg/ml each), incubated for 4 h at 25 °C in the dark, and Alpha detection was carried out (Fig. 2f).

### Titration of the concentration of ATP in the PCNA ubiquitination cascade

Reactions were incubated in the presence of different concentrations of ATP, with the assay carried out under final optimized conditions as above (Additional file 6: Fig. S6a).

### DMSO tolerance of the PCNA ubiquitination cascade

Reactions were incubated in the presence of different concentrations of DMSO, with the assay carried out under final optimized conditions as above (Additional file 6: Fig. S6b).

### Alpha assay for Uba1~ubiquitin thioester formation

Concentrations of FLAG-Uba1 with biotin-ubiquitin concentration held constant at 150 nM (Fig. 3a) were varied, as were biotin-ubiquitin with FLAG-Uba1 concentration held constant at 50 nM (Fig. 3b), under otherwise identical reaction and assay conditions in a buffer consisting of 40 mM Tris-HCl, pH 7.5, 8 mM MgCl_2_, and 10% glycerol. The reactions were initiated by addition of ATP to 2 mM for a final volume of 20 μl, then incubated for 30 min at 25 °C, with reaction termination and dilution (except for the omission of DTT), incubation, and Alpha detection carried out under final optimized conditions as above.

### Alpha assay for Rad6~ubiquitin thioester formation

Concentrations of FLAG-Rad6 with biotin-ubiquitin held constant at 150 nM (Fig. 3c) and biotin-ubiquitin with FLAG-Rad6 held constant at 50 nM (Fig. 3d) were each separately varied, under otherwise identical reaction and assay conditions in a buffer of 40 mM Tris-HCl, pH 7.5, 8 mM MgCl_2_, and 10% glycerol. The Uba1 concentration was 50 nM in these experiments. The reactions were initiated by the addition of 2 mM ATP for a final volume of 20 μl, incubated for 30 min at 25 °C, with termination and dilution (except for the omission of DTT), incubation, and Alpha detection carried out under final optimized conditions as above.

Once satisfactory starting conditions were found, the overall reaction was then conducted in two separate steps, which allows for screening for direct inhibitors of Rad6~ubiquitin thioester formation whose mechanism is not just secondary to inhibition of Uba1~ubiquitin thioester formation (Additional file 7: Fig. S7a). For initial precharging of Uba1 with biotin-ubiquitin, 100 nM Uba1 was combined with 300 nM biotin-ubiquitin, with the reaction initiated by addition of ATP to 2 mM, followed by incubation for 30 min at 25 °C. The reaction sample was then combined with an equal volume of 100 nM FLAG-Rad6 (which can be preincubated with compounds for screening for direct Rad6 inhibitors), for final concentrations of 50 nM Uba1, 150 nM biotin-ubiquitin, and 50 nM FLAG-Rad6, with a final volume of 20 μl. The mixture was incubated for another 30 min at 25 °C, with the remaining procedures as before. To prevent the possibility of further charging of Uba1 with biotin-ubiquitin before addition of Rad6, we performed an experiment where the Uba1 sample was quenched with EDTA prior to mixing with Rad6 (to a final EDTA concentration of 20 mM, a concentration which for some reason negatively affected the transthioesterification reaction despite this step of the cascade not requiring Mg^2+^-ATP, as noted above), with protein concentrations and incubation times being the same (Additional File 7: Fig. S7a). Lower concentrations of ATP (250 μM) and MgCl_2_ (500 μM), with varying concentrations of EDTA (500 μM–8 mM), were also examined, with other conditions being the same (Additional File 7: Fig. S7b).

### Alpha assay for Rad18 autoubiquitination

Concentrations of FLAG-Rad18 with biotin-ubiquitin held constant at 150 nM (Fig. 3f) and concentrations of biotin-ubiquitin with FLAG-Rad18 held constant at 50 nM (Fig. 3g) were each separately varied under otherwise identical reaction and assay conditions in a buffer of 40 mM Tris-HCl, pH 7.5, 8 mM MgCl_2_, and 10% glycerol in a volume of 20 μl. Uba1 was held constant at 50 nM, and Rad6–Rad18 dimer was held at 100 nM. The reaction was incubated at 25 °C for 1 h in Alpha buffer (which includes DTT in it) containing donor and acceptor beads at 10 μg/ml each, and the assay was carried out under final optimized conditions as above.

We applied this two-part procedure also to the Rad18 autoubiquitination assay, wherein we allowed for charging of 100 nM Uba1 and 200 nM biotin-ubiquitin with 250 μM ATP and 500 μM MgCl_2_ for 30 min at 25 °C, then quenched the reaction by addition of EDTA to a final concentration of 1 mM. We mixed the sample with an equal volume of 200 nM Rad6–Rad18 dimer and 100 or 200 nM FLAG-Rad18, as indicated (for final protein concentrations of 50 nM Uba1, 100 nM biotin-ubiquitin, 100 nM Rad6–Rad18 dimer, and 50 or 100 nM FLAG-Rad18), then incubated for an additional 1 h at 25 °C in Alpha buffer containing donor and acceptor beads at 10 μg/ml each and 20 mM EDTA, and the assay was then carried out under final optimized conditions as above (Additional file 7: Fig. S7c).

### Testing of previously identified inhibitors of ubiquitination in the Alpha assay

We employed the above optimized conditions and examined the effect of green tea compounds that we previously identified as inhibitors of ubiquitination at the level of Uba1 [19]. Compounds were added to wells in 96-well plates containing all the components of the reaction except for ATP, with preincubation for 15 min. ATP was then added to 2 mM to initiate the ubiquitination cascade with a final volume of 20 μl, and the procedures for reaction and Alpha assay were the optimized ones described above (Fig. 6). We also conducted Alpha assays for Uba1~ubiquitin thioester formation, with preincubation for 15 min with the compounds prior to initiation of reaction and assay, as described above (Fig. 7a and b).

### Statistical analysis

Statistical analysis was performed with Microsoft Excel and GraphPad Prism 8 software.

## Supplementary information

**Additional file 1: Figure S1.** PCNA ubiquitination reaction volumes. PCNA ubiquitination was conducted in different volumes, followed by incubation in Alpha buffer with donor and acceptor beads and detection. Data represent mean and SD for 8 samples. For conditions and procedures for this and subsequent figures, see Methods.

**Additional file 2: Figure S2.** Dilution factors for detection of ubiquitinated PCNA. PCNA ubiquitination reactions were diluted to different degrees as indicated in Alpha buffer with donor and acceptor beads, followed by incubation and detection. Data represent mean and SD for ≥3 samples. Top and bottom panels represent two separate experiments with different ranges of dilution.

**Additional file 3: Figure S3.** Alpha donor and acceptor bead concentrations and ratios for detection of ubiquitinated PCNA. **a** Concentrations of donor and acceptor beads were varied, as indicated, followed by incubation and detection. Data represent mean and SD for 4 samples **b** Ratios of donor and acceptor beads (values in μg/ml) were varied, followed by incubation and detection. Data represent mean and SD for 7–8 samples. D = donor beads; A = acceptor beads.

**Additional file 4: Figure S4.** Donor and acceptor bead order of addition for detection of ubiquitinated PCNA. The order of addition of Alpha donor and acceptor beads was examined, with incubation for 2 h with one and then further for 2 h after addition of the other (compared to simultaneous addition and incubation for 2 h or 4 h), followed by detection. Data represent mean and SD for 3 samples. D = donor beads; A = acceptor beads.

**Additional file 5: Figure S5.** Donor and acceptor bead incubation times for detection of ubiquitinated PCNA. Times of incubation with beads prior to Alpha detection were evaluated. Data represent mean and SD for 3 samples. **a** Incubation time course with donor and acceptor bead concentrations at 20 μg/ml each. **b** Incubation time course with donor and acceptor bead concentrations at 10 μg/ml each for Alpha detection after PCNA ubiquitination reaction in the presence and absence of ATP, the latter to test whether any non-specific precipitation or other effects would by themselves influence subsequent signal over time. D = donor beads; A = acceptor beads.

**Additional file 6: Figure S6.** Variation of ATP concentration and DMSO tolerance in Alpha assay for PCNA ubiquitination. **a** ATP concentrations for the PCNA ubiquitination cascade were varied, followed by incubation and detection. Data represent mean and SD for 3 samples. **b** Different concentrations of DMSO were added to the reactions, followed by incubation and detection. Data represent mean and SD for 3 samples.

**Additional file 7: Figure S7.a** Split two-part Rad6~ubiquitin thioester formation assay, with precharging of Uba1 with biotin-ubiquitin prior to addition of Rad6 and quenching of Uba1 charging with EDTA to a final concentration of 20 mM (added between steps to chelate Mg^2+^ and prevent further ATP-dependent Uba1 charging with ubiquitin); negative control was without ATP, while the positive control and EDTA-treated samples included ATP. Data represent mean and SD for 8 samples. **b** Modified two-part Rad6~ubiquitin thioester formation assay reaction with 250 μM ATP and 500 μM MgCl_2_, with Uba1 quenching with varying concentrations of EDTA. Data represent mean and SD for 3 samples. **c** Two-part Rad18 autoubiquitination assay with 250 μM ATP and 500 μM MgCl_2_, with Uba1 charging quenched by adding EDTA to 1 mM, followed by addition of 100 nM Rad6–Rad18 dimer and 50 nM or 100 nM FLAG-Rad18. Data represent mean and SD for 4 samples.

**Additional file 8: Figure S8.** SDS–polyacrylamide gel of proteins used in this study. Proteins were resolved on a 12% SDS–polyacrylamide gel and stained with Coomassie Brilliant Blue R-250. Each lane represents a different protein preparation, and the relevant protein bands are indicated with arrow marks. Note: The 5 subunits of *S. cerevisae* RFC were only resolved into 3 bands, since the molecular weights of the larger of the three of the smaller subunits are so close to each other.

## Data Availability

Data and materials used in the current study are available from the corresponding authors on request.

## Abbreviations

Alpha: Amplified luminescent proximity homogeneous assay
CI: Confidence interval
DMSO: Dimethyl sulfoxide
DTT: Dithiothreitol
E1: Ubiquitin-activating enzyme
E2: Ubiquitin-conjugating enzyme
E3: Ubiquitin ligase
EDTA: Ethylenediaminetetraacetic acid
ELISA: Enzyme-linked immunosorbent assay
GST: Glutathione *S*-transferase
IC_50_: Half-maximal inhibitory concentration
NTA: Nitrilotriacetic acid
OD_600_: Optical density at 600 nm
PBS: Phosphate-buffered saline
PCNA: Proliferating cell nuclear antigen
RET: Resonance energy transfer
RFC: Replication factor C
SD: Standard deviation
SE: Standard error
SDS: Sodium dodecyl sulfate
TLS: Translesion DNA synthesis
UBL: Ubiquitin-like protein

## References

[1] Knobel PA, Marti TM (2011). Translesion DNA synthesis in the context of cancer research. Cancer Cell Int.

[2] Sharma S, Helchowski CM, Canman CE (2013). The roles of DNA polymerase ζ and the Y family DNA polymerases in promoting or preventing genome instability. Mutat Res.

[3] Korzhnev DM, Hadden MK (2016). Targeting the translesion synthesis pathway for the development of anti-cancer chemotherapeutics. J Med Chem.

[4] Jansen JG, Tsaalbi-Shtylik A, de Wind N (2015). Roles of mutagenic translesion synthesis in mammalian genome stability, health and disease. DNA Repair (Amst).

[5] Zafar MK, Eoff RL (2017). Translesion DNA synthesis in cancer: molecular mechanisms and therapeutic opportunities. Chem Res Toxicol.

[6] Yang Y, Gao Y, Zlatanou A, Tateishi S, Yurchenko V, Rogozin IB (2018). Diverse roles of RAD18 and Y-family DNA polymerases in tumorigenesis. Cell Cycle.

[7] Dieckman LM, Freudenthal BD, Washington MT (2012). PCNA structure and function: insights from structures of PCNA complexes and post-translationally modified PCNA. Subcell Biochem.

[8] Choe KN, Moldovan GL (2017). Forging ahead through darkness: PCNA, still the principal conductor at the replication fork. Mol Cell.

[9] Slade D. Maneuvers on PCNA rings during DNA replication and repair. Genes (Basel). 10.3390/genes9080416 PMC6116012 30126151

[10] Kanao R, Masutani C (2017). Regulation of DNA damage tolerance in mammalian cells by post-translational modifications of PCNA. Mutat Res.

[11] Leung W, Baxley R, Moldovan G-L, Bielinsky A-K (2018). Mechanisms of DNA damage tolerance: Post-translational regulation of PCNA. Genes (Basel).

[12] Miyase S, Tateishi S, Watanabe K, Tomita K, Suzuki K, Inoue H (2005). Differential regulation of Rad18 through Rad6-dependent mono- and polyubiquitination. J Biol Chem.

[13] Zeman MK, Lin JR, Freire R, Cimprich KA (2014). DNA damage-specific deubiquitination regulates Rad18 functions to suppress mutagenesis. J Cell Biol.

[14] Dove KK, Klevit RE (2017). RING-between-RING E3 ligases: emerging themes amid the variations. J Mol Biol.

[15] Zheng N, Shabek N (2017). Ubiquitin ligases: structure, function, and regulation. Annu Rev Biochem.

[16] Walden H, Rittinger K (2018). RBR ligase–mediated ubiquitin transfer: a tale with many twists and turns. Nat Struct Mol Biol.

[17] Weber J, Polo S, Maspero E (2019). HECT E3 ligases: a tale with multiple facets. Front Physiol.

[18] Eglen RM, Reisine T, Roby P, Rouleau N, Illy C, Bossé R (2008). The use of AlphaScreen technology in HTS: current status. Curr Chem Genomics.

[19] Fenteany G, Gaur P, Hegedűs L, Dudás K, Kiss E, Wéber E (2019). Multilevel structure–activity profiling reveals multiple green tea compound families that each modulate ubiquitin-activating enzyme and ubiquitination by a distinct mechanism. Sci Rep.

[20] Kus B, Gajadhar A, Stanger K, Cho R, Sun W, Rouleau N (2005). A high throughput screen to identify substrates for the ubiquitin ligase Rsp5. J Biol Chem.

[21] Rouleau N, Wang J, Karras L, Andrews E, Bielefeld-Sevigny M, Chen Y (2008). Highly sensitive assays for SUMOylation and small ubiquitin-like modifier-dependent protein–protein interactions. Anal Biochem.

[22] Takahashi H, Nozawa A, Seki M, Shinozaki K, Endo Y, Sawasaki T (2009). A simple and high-sensitivity method for analysis of ubiquitination and polyubiquitination based on wheat cell-free protein synthesis. BMC Plant Biol.

[23] Schneider S, Chen H, Tang J, Emkey R, Andrews PS (2012). Development of a homogeneous AlphaLISA ubiquitination assay using ubiquitin binding matrices as universal components for the detection of ubiquitinated proteins. Biochim Biophys Acta, Mol Cell Res.

[24] Yan Z-H, Burkhardt A, Loke H-K, Chen J, Xu Q, Brauer P (2013). Quantifiable analysis of cellular pathway inhibition of a Nedd8-activating enzyme inhibitor, MLN4924, using AlphaScreen. Anal Biochem.

[25] Li YJ, Du L, Wang J, Vega R, Lee TD, Miao Y (2019). Allosteric inhibition of ubiquitin-like modifications by a class of inhibitor of SUMO-activating enzyme. Cell Chem Biol.

[26] Finkelstein J, Antony E, Hingorani MM, O’Donnell M (2003). Overproduction and analysis of eukaryotic multiprotein complexes in Escherichia coli using a dual-vector strategy. Anal Biochem.

[27] Haracska L, Unk I, Prakash L, Prakash S (2006). Ubiquitylation of yeast proliferating cell nuclear antigen and its implications for translesion DNA synthesis. Proc Natl Acad Sci U S A.

[28] Unk I, Hajdú I, Fátyol K, Hurwitz J, Yoon J-H, Prakash L (2008). Human HLTF functions as a ubiquitin ligase for proliferating cell nuclear antigen polyubiquitination. Proc Natl Acad Sci U S A.

[29] Juhasz S, Balogh D, Hajdu I, Burkovics P, Villamil MA, Zhuang Z (2012). Characterization of human Spartan/C1orf124, an ubiquitin–PCNA interacting regulator of DNA damage tolerance. Nucleic Acids Res.

[30] Gomes XV, Gary SL, Burgers PM (2000). Overproduction in Escherichia coli and characterization of yeast replication factor C lacking the ligase homology domain. J Biol Chem.

