# Supplementary figures and images for: Robust high-throughput assays to assess discrete steps in ubiquitination and related cascades
Source: BMC Mol Cell Biol. 2020 Mar 30;21:21. doi: 10.1186/s12860-020-00262-5 (PMC7106726; doi:10.1186/s12860-020-00262-5)

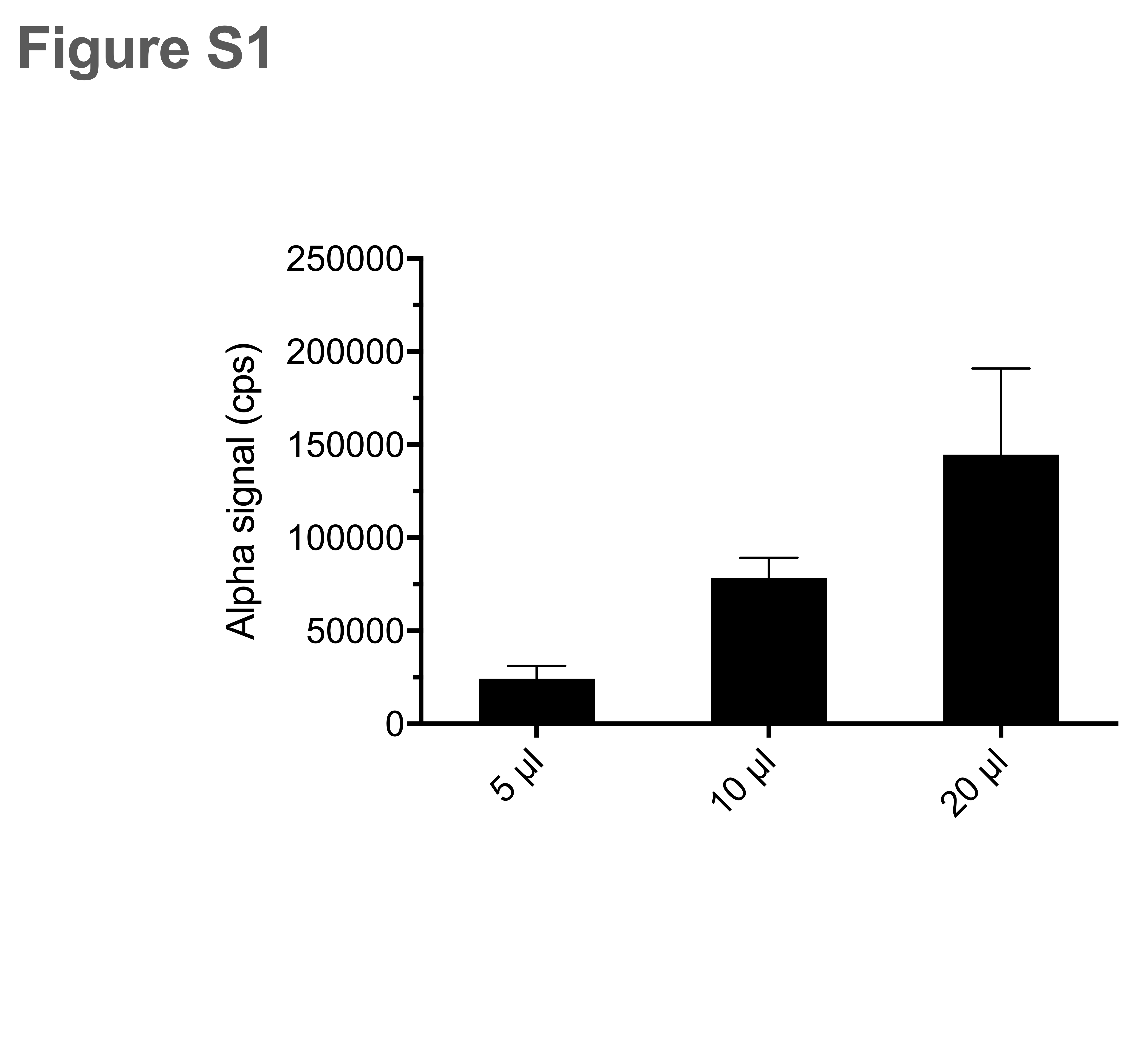

Supplement: Supplementary file 1 — Additional file 1: Figure S1. PCNA ubiquitination reaction volumes. PCNA ubiquitination was conducted in different volumes, followed by incubation in Alpha buffer with donor and acceptor beads and detection. Data represent mean and SD for 8 samples. For conditions and procedures for this and subsequent figures, see Methods. [file 12860_2020_262_MOESM1_ESM.tiff]

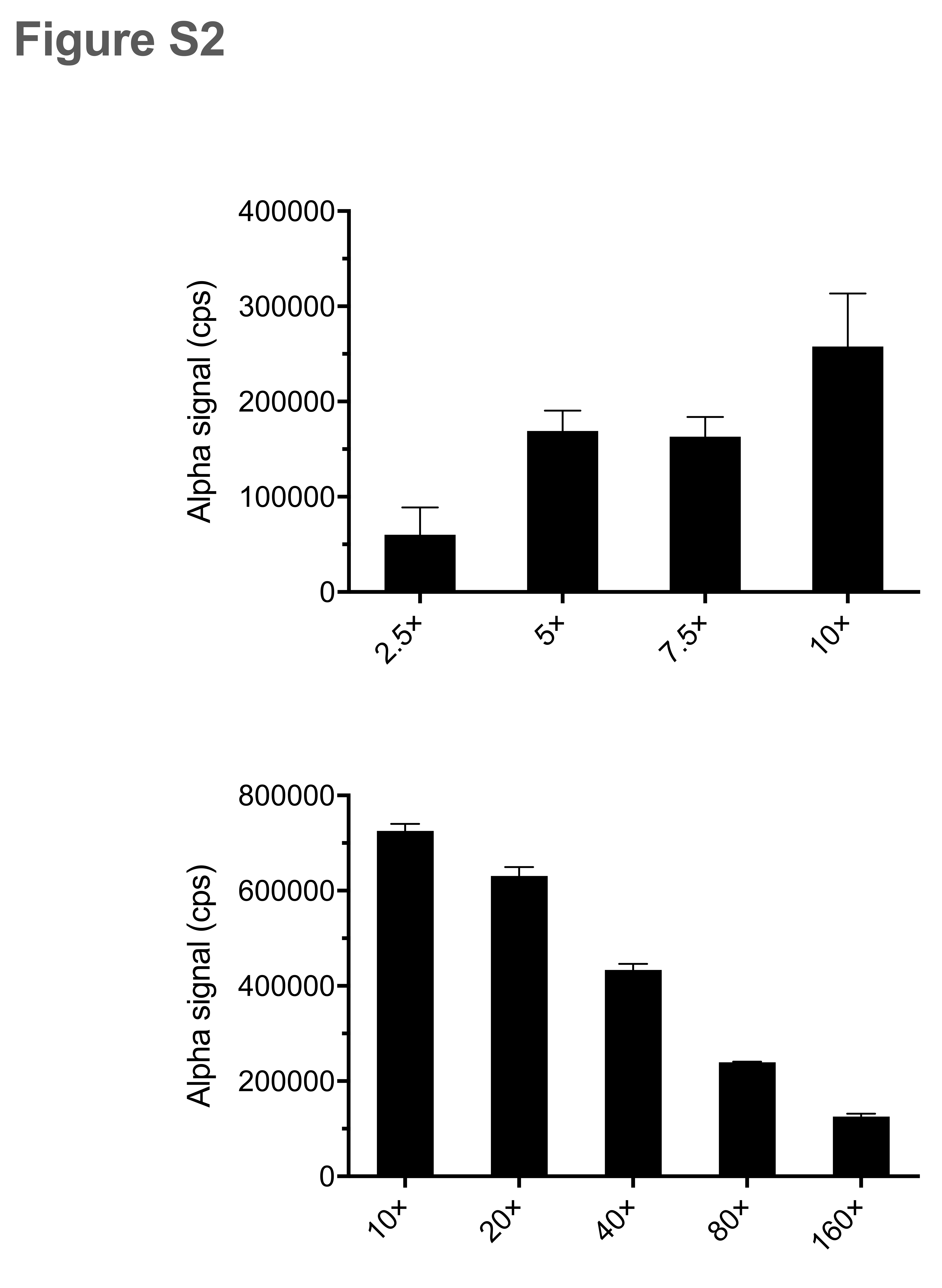

Supplement: Supplementary file 2 — Additional file 2: Figure S2. Dilution factors for detection of ubiquitinated PCNA. PCNA ubiquitination reactions were diluted to different degrees as indicated in Alpha buffer with donor and acceptor beads, followed by incubation and detection. Data represent mean and SD for ≥3 samples. Top and bottom panels represent two separate experiments with different ranges of dilution. [file 12860_2020_262_MOESM2_ESM.tiff]

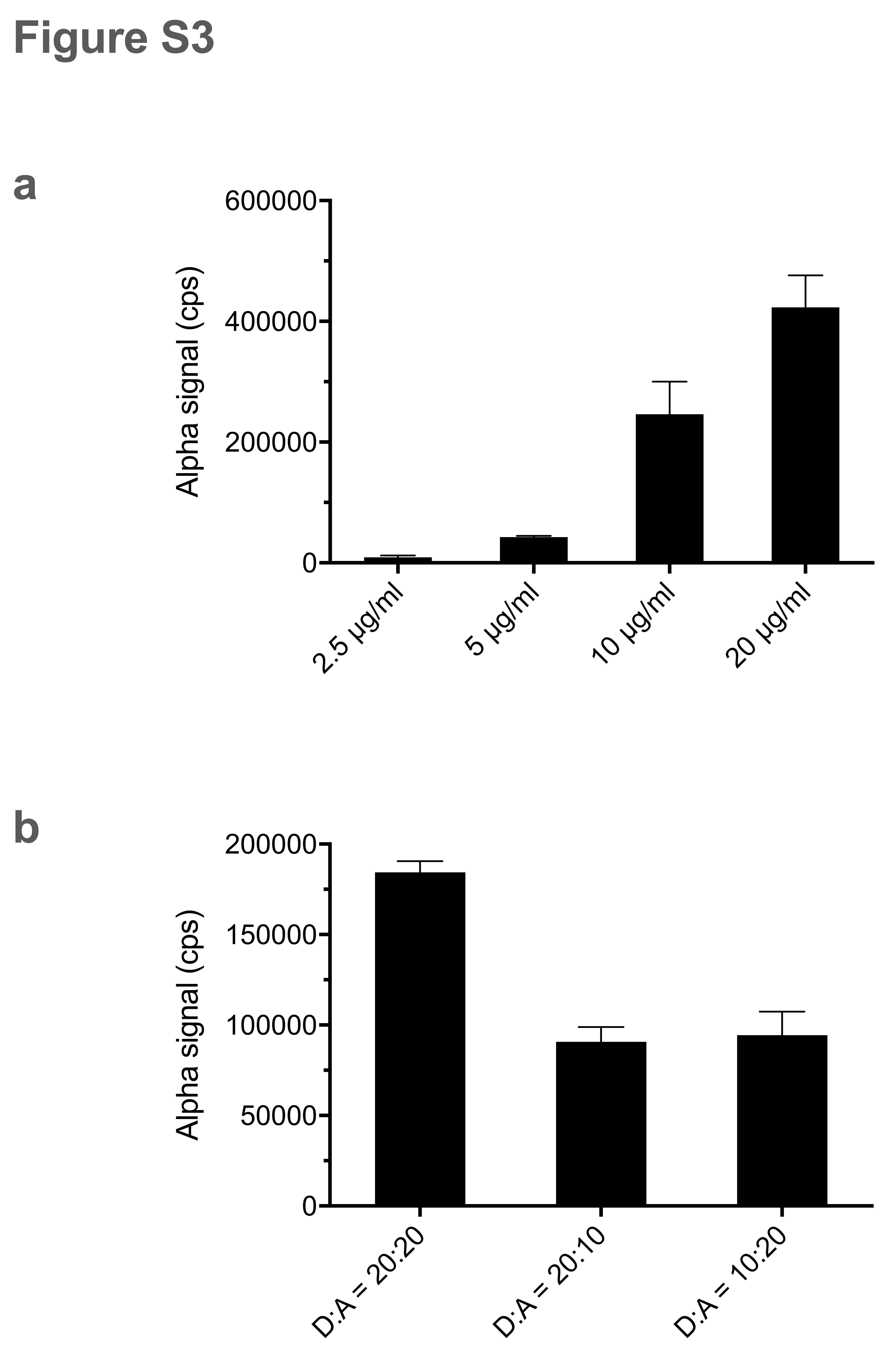

Supplement: Supplementary file 3 — Additional file 3: Figure S3. Alpha donor and acceptor bead concentrations and ratios for detection of ubiquitinated PCNA. a Concentrations of donor and acceptor beads were varied, as indicated, followed by incubation and detection. Data represent mean and SD for 4 samples b Ratios of donor and acceptor beads (values in μg/ml) were varied, followed by incubation and detection. Data represent mean and SD for 7–8 samples. D = donor beads; A = acceptor beads. [file 12860_2020_262_MOESM3_ESM.tiff]

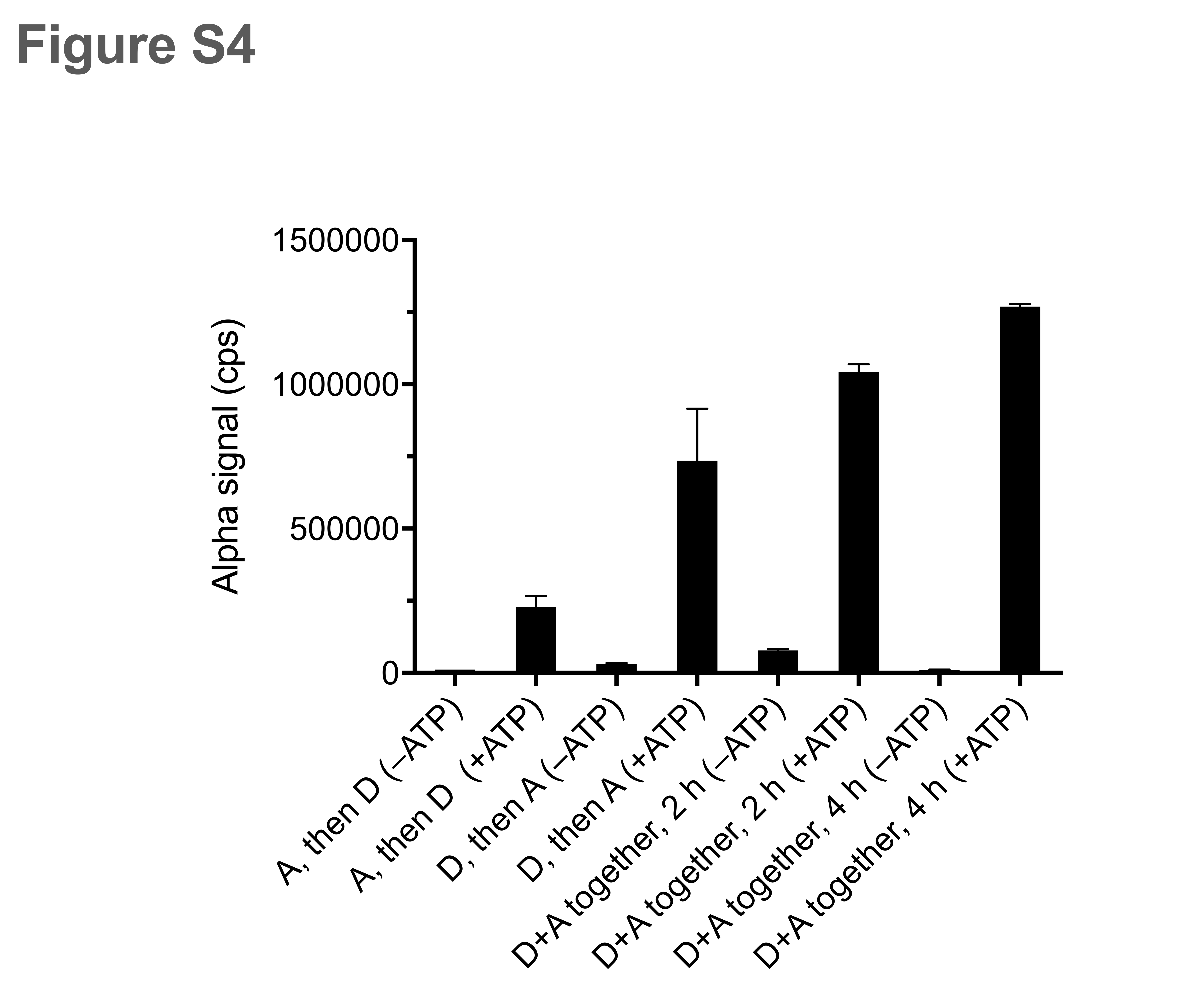

Supplement: Supplementary file 4 — Additional file 4: Figure S4. Donor and acceptor bead order of addition for detection of ubiquitinated PCNA. The order of addition of Alpha donor and acceptor beads was examined, with incubation for 2 h with one and then further for 2 h after addition of the other (compared to simultaneous addition and incubation for 2 h or 4 h), followed by detection. Data represent mean and SD for 3 samples. D = donor beads; A = acceptor beads. [file 12860_2020_262_MOESM4_ESM.tiff]

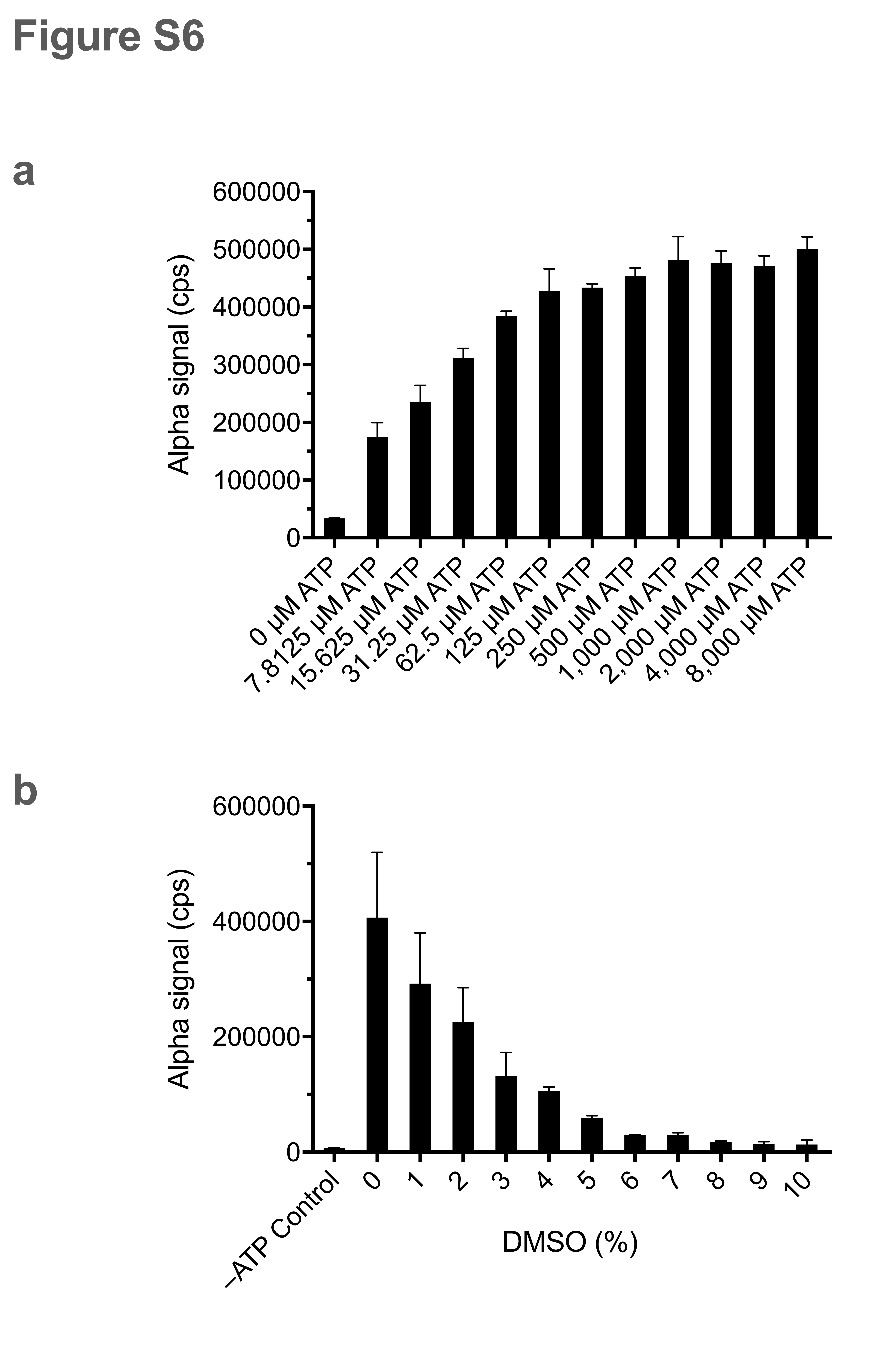

Supplement: Supplementary file 6 — Additional file 6: Figure S6. Variation of ATP concentration and DMSO tolerance in Alpha assay for PCNA ubiquitination. a ATP concentrations for the PCNA ubiquitination cascade were varied, followed by incubation and detection. Data represent mean and SD for 3 samples. b Different concentrations of DMSO were added to the reactions, followed by incubation and detection. Data represent mean and SD for 3 samples. [file 12860_2020_262_MOESM6_ESM.tiff]

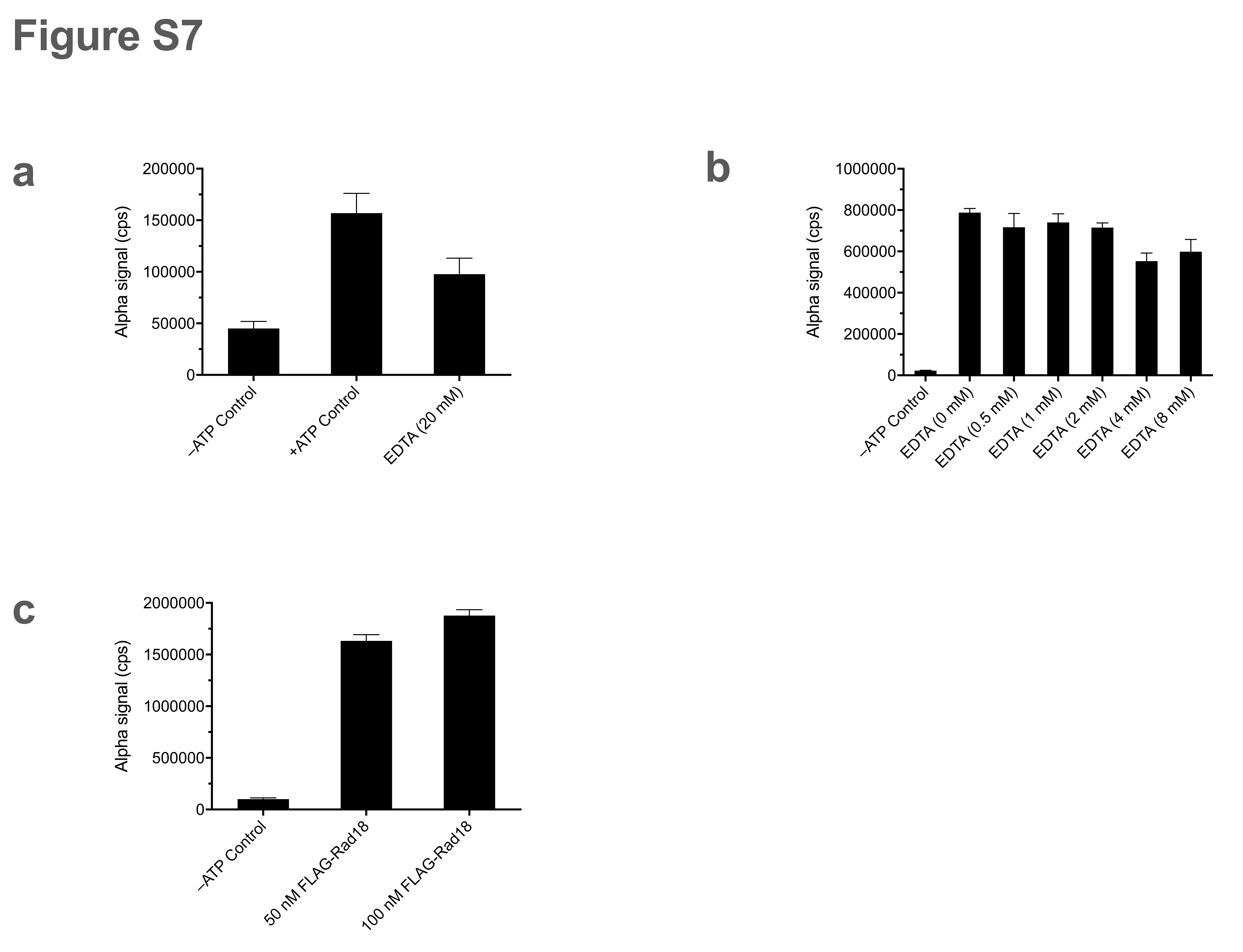

Supplement: Supplementary file 7 — Additional file 7: Figure S7.a Split two-part Rad6~ubiquitin thioester formation assay, with precharging of Uba1 with biotin-ubiquitin prior to addition of Rad6 and quenching of Uba1 charging with EDTA to a final concentration of 20 mM (added between steps to chelate Mg2+ and prevent further ATP-dependent Uba1 charging with ubiquitin); negative control was without ATP, while the positive control and EDTA-treated samples included ATP. Data represent mean and SD for 8 samples. b Modified two-part Rad6~ubiquitin thioester formation assay reaction with 250 μM ATP and 500 μM MgCl2, with Uba1 quenching with varying concentrations of EDTA. Data represent mean and SD for 3 samples. c Two-part Rad18 autoubiquitination assay with 250 μM ATP and 500 μM MgCl2, with Uba1 charging quenched by adding EDTA to 1 mM, followed by addition of 100 nM Rad6–Rad18 dimer and 50 nM or 100 nM FLAG-Rad18. Data represent mean and SD for 4 samples. [file 12860_2020_262_MOESM7_ESM.tiff]

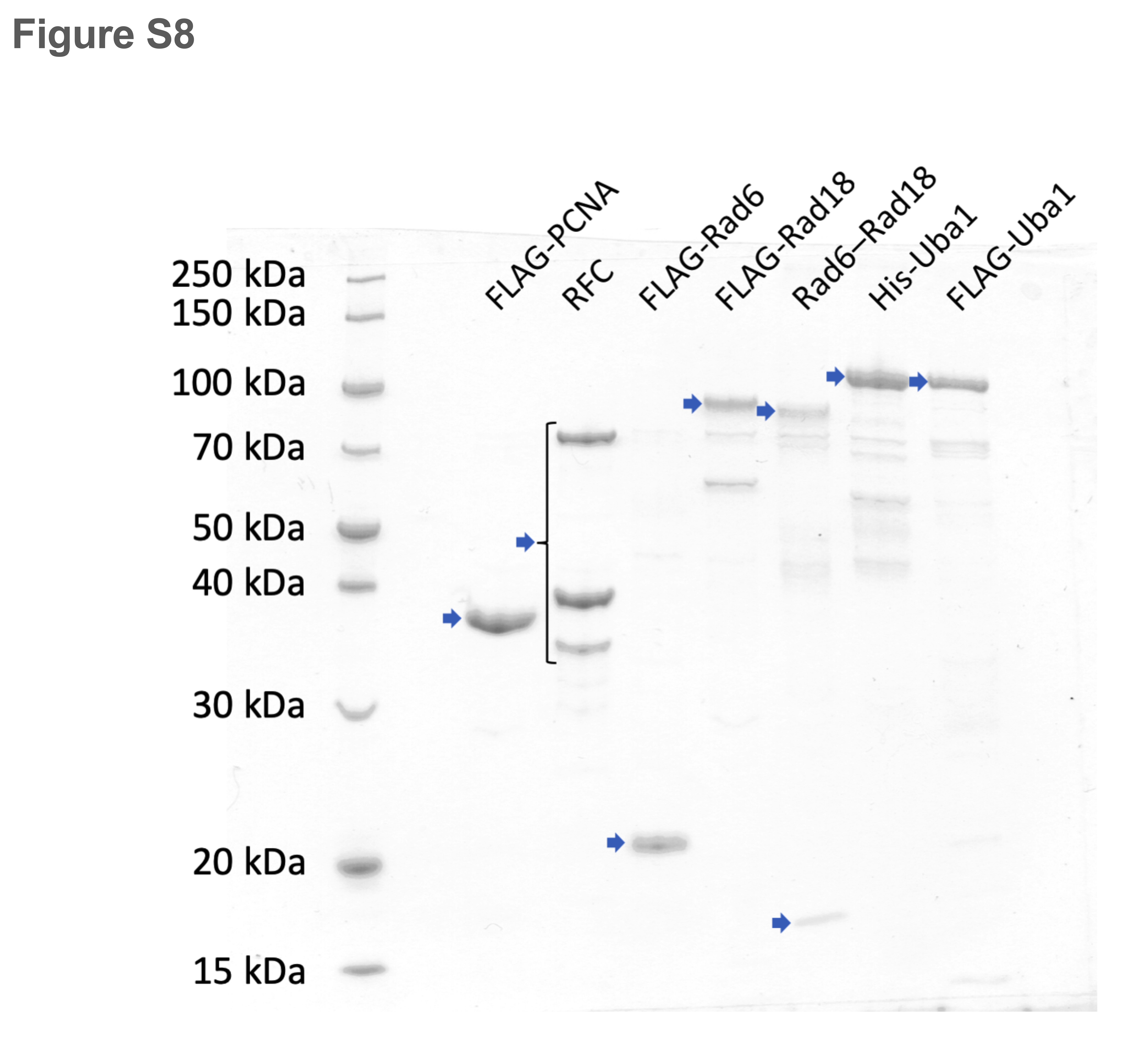

Supplement: Supplementary file 8 — Additional file 8: Figure S8. SDS–polyacrylamide gel of proteins used in this study. Proteins were resolved on a 12% SDS–polyacrylamide gel and stained with Coomassie Brilliant Blue R-250. Each lane represents a different protein preparation, and the relevant protein bands are indicated with arrow marks. Note: The 5 subunits of S. cerevisae RFC were only resolved into 3 bands, since the molecular weights of the larger of the three of the smaller subunits are so close to each other. [file 12860_2020_262_MOESM8_ESM.tiff]
